# Semantic and Phonological Brain Networks in Older Adults: A Systematic Scoping Review

**DOI:** 10.3390/brainsci16030252

**Published:** 2026-02-25

**Authors:** Victoria A. Diedrichs, David E. Osher, Stacy M. Harnish, Erin L. Meier

**Affiliations:** 1 Department of Communicative Sciences and Disorders, College of Communication Arts and Sciences, Michigan State University, East Lansing, MI 48824, USA; 2 Shirley Ryan AbilityLab, Chicago, IL 60611, USA; 3 Department of Psychology, College of Arts and Sciences, The Ohio State University, Columbus, OH 43210, USA; 4 Department of Speech and Hearing Science, College of Arts and Sciences, The Ohio State University, Columbus, OH 43210, USA; 5 Department of Communication Sciences and Disorders, Bouvé College of Health Sciences, Northeastern University, Boston, MA 02115, USA

**Keywords:** language, semantics, phonology, cognition, aging

## Abstract

**Highlights:**

**What are the main findings?**

- In our scoping review of older adult studies, we found similarities with regions implicated in semantic and phonological processing in younger adults.
- Regions with the potential for semantic and phonological specialization in older adults were largely confined to the ventral and dorsal stream pathways, respectively.

**What are the implications of the main findings?**

- The results of this scoping review are generally compatible with the CRUNCH or de-differentiation hypothesis.
- Complementary activation- and lesion-based meta-analyses in older adults are recommended.

**Abstract:**

**Background/Objectives**: Despite many neurobiological models of semantics and phonology, there is no consensus regarding their functional organization. Specifically, there is considerable debate concerning the precise functional roles of the left inferior frontal gyrus and inferior parietal lobe, where these two networks may overlap. Meta-analyses addressing this topic have focused on neurologically intact young adults, yet theories of cognitive aging suggest older adults may exhibit a different distribution of their language networks. This scoping review specifically explores the interplay between semantic and phonological neural networks in older adults. **Methods**: Following the PRISMA extension guidelines, we carried out a systematic search to identify relevant primary research. Thirty-seven studies were included, representing a range of populations (e.g., neurologically intact older adults, post-stroke aphasia), methodologies (e.g., task-based functional magnetic resonance imaging, lesion symptom mapping), and sample sizes from 11 to 1231 participants. **Results**: Based on the number of studies identifying relationships with a given region, we found that regions potentially specialized for semantics or phonology and those potentially subserving both domains are largely consistent with networks derived from meta-analyses of younger adults. However, we found subtle differences between older and younger adults. **Conclusions**: Our results are generally compatible with the CRUNCH or de-differentiation perspectives of cognitive aging. In our characterization of the literature related to the semantic and phonological networks of older adults, we did not see a pattern consistent with the HAROLD or PASA models, though our findings were limited by methodological decisions. Our work suggests that future complementary activation- and lesion-based meta-analyses are warranted to more precisely delineate the semantic and phonological networks in older adults.

## 1. Introduction

The neural correlates of language have been extensively studied, and there have been many attempts to model the organization of language systems in the brain. However, there remains disagreement about this organization. Although attempts have been made to reconcile findings through the use of meta-analyses, among other approaches, these have largely focused on younger neurologically intact adults. As a result, less is known about the organization of language in the brains of older adults, which is important at a basic level, for our understanding of age-related cognitive changes, and at a clinical level, for populations whose language may be affected at an older age (e.g., post-stroke aphasia, primary progressive aphasia). To address these gaps, the present scoping review will explore the neural correlates of two language domains, semantics and phonology, in older adults, with particular attention to their points of overlap.

### 1.1. Overview of Models of Semantics and Phonology

Broadly, semantic processing refers to the use, storage, and retrieval of multimodal conceptual information, whereas phonological processing refers to the use, storage, perception, and analysis of linguistic sounds. Tasks that tax the semantic system may involve word retrieval for items in a given category, synonym judgment, spoken or written word to picture matching, and even reading irregularly spelled words. Likewise, tasks that tax the phonological system may involve word retrieval for items beginning with the same phoneme, rhyme judgment, minimal pair discrimination, and reading or repeating nonwords. Here, we present a brief overview of models endeavoring to explain the spatial organization of semantics and phonology in the brain. Our goal is not to provide a detailed account of any one model but instead to highlight discrepancies between prominent and influential models that raise questions related to the integration of semantic and phonological networks. Moreover, we will use these models to contextualize our own results in the discussion.

One of the most influential and widely cited models of the language network is the dual-stream model by Hickok and Poeppel [1,2], which organizes speech processing similarly to models for other sensory systems in the brain (e.g., vision), including a dorsal stream mapping sound onto articulatory-based representations and a ventral stream mapping sound onto meaning. In this model, the authors propose that following spectrotemporal analysis and phonological processing in the bilateral superior temporal lobe, the dorsal pathway maps sensory or phonological representations onto articulatory motor representations in left insular and frontal cortices (including Broca’s area, traditionally consisting of Brodmann areas [BA] 44 and 45) via an area referred to as Spt within the planum temporale at the left temporoparietal junction. Meanwhile, the ventral pathway maps sensory or phonological representations onto lexical conceptual representations throughout segments of the left and bilateral temporal cortex. Another model of the language network by Friederici and Gierhan [3] roughly aligns with the dual-stream model, although it includes the anterior inferior frontal cortex (i.e., BA 45 and 47, frontal operculum) in the ventral stream and emphasizes the role of regions within each stream in syntactic processing. Moreover, unlike Hickok and Poeppel [1,2], Friederici and Gierhan [3] specify the white matter association tracts that connect important cortical regions in the brain: the arcuate and superior longitudinal fasciculi represent the structural dorsal pathways, whereas the uncinate and inferior fronto-occipital fasciculi represent the structural ventral pathways.

A competing attempt to model language in the brain is Hagoort’s Memory, Unification, and Control model [4,5], which departs significantly from the organizational structure of the previous two models. For the memory component of the MUC model [4,5], Hagoort specifies that phonological/phonetic properties are most likely to be stored in the central-to-posterior superior temporal lobe; semantic information, on the other hand, is likely to be distributed across the middle and inferior temporal gyri and angular gyrus. The generative process of creating new meaning from these linguistic building blocks (i.e., unification) takes place within BA 47 and 45 for semantics and within BA 44 and parts of BA 6 for phonology. Finally, unlike the dual-stream model, Hagoort [4,5] emphasizes that language is related to joint action and social interaction through selection of the appropriate targets (i.e., control) within left dorsolateral prefrontal and anterior cingulate cortex.

In addition to the relatively comprehensive language models discussed above, several influential models of semantics exist, including Binder and Desai’s model of semantic memory [6], Lambon Ralph et al.’s model of controlled semantic cognition (combining a graded hub-and-spoke representational network and a semantic control network) [7], Mirman and colleagues’ dual-hub model of taxonomic and thematic systems [8], and Xu et al.’s tri-network model of semantic processing [9]. Overall, there are fewer existing models of phonology than semantics, which may contribute to the lack of consensus on the spatial distribution of these two networks. Two models emphasize lexical phonology: an updated model of spatial and temporal signatures of word production [10,11] and a shared model of pseudoword repetition and picture-naming processes [12].

A key inconsistency between these collective models is the specific functional role of subdivisions within the inferior frontal gyrus, particularly between anterior and posterior parcellations of this region. For example, as a component of Broca’s area, the inferior frontal gyrus, pars triangularis (BA 45), is part of Hickok and Poeppel’s articulatory network within the dorsal stream [1,2] and is proposed to play a role in syllabification by Indefrey [10]. However, BA 45 also appears as a site of semantic (and syntactic) unification in Hagoort’s MUC model [4,5], and several other models propose that it is involved in semantic control and selection processes rather than downstream phonological or articulatory processes [3,6,7,9]. While pars orbitalis is more often implicated in semantics [3,4,6,9] and pars opercularis is more often implicated in phonological processes [2,4,10], debate remains regarding those parcels, and the role of pars triangularis is especially unclear.

Another point of contention between proposals of the neural architecture of semantics and phonology is the role of the inferior parietal lobe versus the temporoparietal junction. Indefrey broadly implicates the inferior parietal lobe in word production, suggesting that a more specific role is unclear [10]. Graves et al. similarly present ambiguous evidence for its potential role in phonetic encoding and articulation or word learning and phonological working memory [12]. Notably, Indefrey [10] and Graves et al. [12] refer to the inferior parietal lobe broadly in their models, without specifying precise roles for its subdivisions. Meanwhile, Hickok and Poeppel [2] propose that the neighboring area Spt in the left planum temporale serves as a sensorimotor interface between their phonological and articulatory networks, yet Binder and Desai [6], Lambon Ralph et al. [7], and Xu et al. [9] argue that the inferior parietal lobe, including the angular and supramarginal gyri especially in the left hemisphere, serves a similar interfacing role in integrating sensory information. However, other models propose different roles for the inferior parietal cortex, such as a specific role for the angular gyrus in lexical and semantic feature representation in the MUC model [4] and for the inferior parietal lobe as a thematic hub in Mirman et al.’s dual-hub model [8]. Taken together, these models offer little clarity on the specific roles of subregions within the inferior parietal lobe and the neighboring temporoparietal junction.

Specific to semantics, one of the central differences between models is whether there exists a hub within the network and which region(s) have this role. Some semantic models support a role for the anterior temporal lobe as an amodal hub for general semantic processing [7,9]. Mirman and colleagues instead propose that the anterior temporal lobe specifically subserves taxonomic associations, while the temporoparietal cortex serves as the hub for thematic associations [8]. Still, other models have no semantic hub [2] or the alternative existence of multiple information convergence zones [6]. Some researchers [13] propose that the organization of concepts and categorical semantic representations are spatially distributed throughout the cortex, driven by the functional connectivity of associated brain regions, which may or may not be compatible with the existence of a semantic hub.

Although the models presented above are not a comprehensive accounting of all attempts to explain the organization of semantic and phonological brain networks, it is clear that, among these influential examples, there is no consensus on the precise functional architecture of these systems. Despite relative agreement regarding the specialization of some brain regions for either semantic or phonological functions, there remains disagreement and ambiguity in specific areas. Importantly, meta-analyses that have leveraged large quantities of neuroimaging data suggest there is also some degree of overlap, roughly consistent with the specific contended areas [14,15,16,17].

As a relevant example, a recent study including a series of activation likelihood estimation meta-analyses [14] directly addressed the question of how dissociable the semantic and phonological brain networks are, detailing the extent of their overlap and distinction in neurologically intact younger adults (i.e., age < 40 in all included primary research articles). This study incorporated data from several existing meta-analyses [15,16,17,18,19]. Activity uniquely contributing to phonology, identified by contrasting it with the semantic results, was found broadly in the left hemisphere at the posterior frontal lobe, inferior parietal lobule, and posterior superior temporal lobe, including clusters within the left precentral gyrus (extending into pars opercularis of the inferior frontal gyrus), supramarginal gyrus (extending into the dorsal angular gyrus), and posterior superior temporal gyrus, as well as smaller clusters in the precuneus and posterior inferior temporal/fusiform gyrus. Activity unique to semantics, contrasted with the phonological results, was found broadly in the left hemisphere at the anterior temporal lobe, medial temporal lobe, and inferior parietal lobule, including clusters within the left anterior middle and superior temporal gyri, parahippocampal and fusiform cortices, and ventral angular gyrus, as well as smaller clusters in the dorsal posterior middle temporal gyrus, superior frontal gyrus, and pars orbitalis of the inferior frontal gyrus.

Highly relevant to the present investigation, Hodgson and colleagues also identified overlap between the semantic and phonological activation likelihood maps, most notably in the left dorsal inferior frontal and posterior inferior temporal gyri [14]. Overlap was also noted in the left precentral gyrus, right inferior frontal gyrus, and bilateral dorsomedial prefrontal cortex. However, the authors revealed through additional meta-analyses of working memory and the multiple-demand network [20] that these regions all play a role in domain-general cognitive control, meaning that the only overlap between the semantic and phonological networks is due to recruiting resources shared with domain-general cognitive functions. The anterior-most part of the inferior frontal gyrus (i.e., pars orbitalis), as well as the posterior middle temporal gyrus, appear to be specialized for semantic-specific control, which is necessary for manipulating activation within the representational semantic system to generate contextually appropriate responses [7]. Given that no regions of overlap were identified between the semantic and phonological representational networks (which encode modality-independent domain-specific knowledge), Hodgson et al. concluded that these representational networks are highly specialized in neurologically healthy young adults [14]. A complicating factor in this work is that most studies mapping the language systems in the brain, including the above meta-analyses, focus on younger, neurologically intact adults. While the organization of the brain likely does not change dramatically during the process of healthy aging, subtle differences between younger and older adults may be expected and could have relevant implications for both healthy older adults and clinical populations whose language may be affected by disease later in life (e.g., stroke survivors with aphasia).

### 1.2. The Impact of Aging on Language and the Brain

It is widely understood that with age comes a gradual decline in cognitive performance due to neuronal loss. This applies to language, although some aspects of language are more affected than others. For example, although older adults demonstrate preservation of lexical semantics and a robust vocabulary, their word retrieval (i.e., picture naming) is slower than younger adults [21]. Broadly, language production appears to be impacted by aging more than language comprehension [22]. Studies have proposed that the degree of language skill preservation, despite age-related atrophy in left-hemisphere language regions, is influenced by the degree to which brain areas not traditionally involved in language processing (e.g., right hemisphere, bilateral frontal regions) are recruited [23,24,25]. These findings are supported by multiple models of age-related brain reorganization, which we will discuss in turn.

In a seminal review by Cabeza [26], it was reported that the most salient difference between the functional brain activity of older and younger adults related to cognition is that older adults are less lateralized than their younger counterparts in the prefrontal cortex. This has been termed the hemispheric asymmetry reduction in older adults (HAROLD) model [26,27] and encompasses the domains of episodic memory, semantic memory, working memory, perception, and inhibitory control. Cabeza cites two different perspectives on why this de-lateralization takes place: a compensation perspective, speculating that bilateral activation helps counteract age-related neurocognitive decline, and a de-differentiation perspective, speculating that older adults struggle to activate highly specialized, lateralized cognitive regions. The underlying cause of age-related changes, including de-lateralization, is still debated [28,29,30] and may vary based on task or difficulty [31]. 

In addition to HAROLD, there are other models of age-related changes in the brain. The compensation-related utilization of neural circuits hypothesis (CRUNCH) proposes that age-related overactivation is compensatory, resulting from the aging brain adapting to its own decline by recruiting resources beyond those required for younger brains to accomplish the same task [31,32]. This hypothesis covers perceptual, motoric, mnemonic, verbal, and spatial domains [31], and the neural sites of overactivation can vary, including contralateral homologues of regions activated by younger adults and bilateral prefrontal cortex, which is associated with processing increasing task demands [32]. As such, CRUNCH is compatible with the HAROLD model and serves to substantiate the compensation perspective for de-lateralization underlying HAROLD.

A third theory accounting for changes in the aging brain is referred to as the posterior–anterior shift in aging (PASA [33]). This theory is also compatible with the others and is consistent with a compensation perspective. PASA originated with a study demonstrating that a decrease in occipital activity among older adults was accompanied by an increase in prefrontal cortex activity during visual processing, suggesting recruitment of higher-order cognitive resources to accomplish the task [34]. Since then, a PASA pattern has been shown in additional studies of visual perception as well as attention and working memory. Notably, Dennis and Cabeza cite evidence that cortical regions supporting core language functions are sufficiently preserved to not require an anterior shift; however, increased working memory or visual perception demands involved in language tasks do result in a PASA pattern [33].

Contrary to the previous hypotheses supporting a compensation account for brain activity unique to older adults (compared with their younger counterparts), a competing de-differentiation account suggests that more widespread activity with age may reflect difficulty sufficiently engaging selective, specialized (e.g., domain-specific) networks; that is, older adults instead engage additional, less selective (e.g., domain-general) networks, which may contribute to impaired functioning [35,36,37]. This view cites age-related increases in correlations across tasks as evidence that older adults regularly recruit a larger area of the brain to accomplish a larger variety of activities [38]. Despite the original view that de-differentiation either does not contribute to or potentially harms task performance, which puts the de-differentiation perspective in direct conflict with the compensation perspective, Dennis and colleagues [35] argue that the less selective networks recruited by older adults may actually facilitate compensation, evidenced by similar behavioral performance in younger adults and older adults who demonstrate a de-differentiated neural pattern [39]. Indeed, others have described that a de-differentiated pattern could be compensatory, if associated with maintained task performance; the key difference between the two perspectives is that de-differentiation involves recruitment of more widespread, generalized resources, whereas compensation typically refers to recruitment of focused areas, specific to the task at hand [25].

The application of these models to the study of age-related changes in activation patterns associated with language processing has produced mixed results [23,24,40,41,42,43,44,45]. For example, in support of the HAROLD model, many studies [23,24,40,42,43,44] report greater right hemisphere activity among older adults compared to younger adults during tasks engaging language skills (e.g., semantics; picture naming or recognition; and sentence recognition, judgment, or processing). Findings from several studies were also compatible with the CRUNCH proposal due to evidence of de-lateralization and recruitment of relatively focused brain regions in response to task demands [24,40,42,44]. However, the results of the meta-analysis by Hoffman and Morcom [43] were consistent with de-differentiation, citing reduced performance associated with de-lateralization and activation of more widespread cognitive regions. Meanwhile, Nenert et al. [41] did not find evidence in support of a consistent HAROLD pattern in their study that specifically examined the lateralization index across participants. These inconsistent findings highlight the need to examine the semantic and phonological networks of older adults, particularly in terms of lateralization.

### 1.3. The Present Scoping Review

Given the collective evidence of distinct semantic and phonological networks in younger adults [14], with overlap at regions associated with domain-general cognitive control, and age-related changes in cognitive network architecture [26,27,31,32,33,36,37], we hypothesize that in older adults, regions associated with semantic and phonological tasks will remain localized to ventral and dorsal regions, respectively, and that older adults will also activate a broader range of bilateral regions than is implicated by Hodgson et al. [14]. To test this hypothesis, we conducted a scoping review with the primary aim of exploring the extent of semantic and phonological brain networks in older adults and the secondary aim of drawing comparisons with the evidence related to the semantic and phonological networks in younger adults. Moreover, as with any scoping review, we also summarize the nature of the evidence reviewed and gauge the potential value in conducting a systematic review or meta-analysis to address this topic. These goals align with the suggested rationale for a scoping review as outlined by Tricco et al. [46].

Based on the purposes outlined above, the results of the scoping review will describe brain regions broadly implicated in semantics, phonology, or both domains. We also specify the number of studies involving neurologically healthy and clinical samples that support the role of each brain region. After describing the nature of the included studies (e.g., sample populations, methods used), we compare our results with the findings of a recent meta-analytic study examining a similar question about semantic and phonological functional organization in exclusively neurologically intact younger adults [14]. Finally, we describe the future directions for this work, with specific attention to the potential for conducting a similar meta-analysis in older adults. Our results may inform future work related to the diagnosis and treatment of acquired language disorders that often occur in older adults and theories of cognitive aging as they relate to language.

## 2. Materials and Methods

Reporting guidelines from the PRISMA extension for scoping reviews (PRISMA-ScR [46]) as well as recommendations from Arksey and O’Malley [47] were used for the present study. An academic librarian was consulted to assist with search strategy development. The systematic search strategy agreed upon by all authors and the librarian was carried out in July 2022 by the first author. Three databases were searched for research articles addressing regions or networks of the brain involved in semantics and phonology: PubMed, Web of Science, and EBSCOhost. Boolean search terms for each database are shown in Table 1.

The following a priori criteria determined whether studies were included: (1) the study consisted of an empirical analysis published in a peer-reviewed journal; (2) the article was written in English; (3) participants had a mean age of 60 or greater; (4) the study included behavioral tasks assessing both semantics and phonology (e.g., category and letter fluency); (5) the analyses included magnetic resonance imaging (MRI) data (e.g., task-based or resting state functional MRI, lesion symptom mapping, and diffusion-weighted or diffusion-tensor imaging) or intraoperative cortical stimulation data; and (6) the relationship between MRI or cortical stimulation data and behavioral performance was statistically analyzed.

Importantly, studies including clinical populations were included, except when the analysis consisted only of fMRI activation, considering such activation may be influenced by lesions, atrophy, reorganization, and/or recovery. In contrast, studies with clinical populations employing various lesion-symptom-mapping approaches were included in the scoping review due to the causal evidence they offer regarding brain–behavior relationships, acting as a complement to functional neuroimaging studies in neurologically intact adults [48]. Reviews, comments, and book chapters that did not present new data were excluded; however, these sources were reviewed to identify additional studies that may have been missing from our search results. Foreign language articles were excluded due to constraints on time and translation costs. The mean age of 60 was utilized as a cut-off based on a prior review that used this mean age to distinguish older adults [43]. Studies that included groups of younger participants were used for the purpose of descriptive comparison in our results (see Section 3.4), but the separate younger adult groups were not incorporated into our primary results addressing older adults. We did not specify or restrict the type of linguistic tasks to be used, other than that they targeted semantics or phonology, as determined by the authors of the original articles and consensus by the first and senior authors of this review. As such, we accepted studies utilizing tasks engaging receptive, expressive, auditory, and visual systems at a variety of linguistic levels (e.g., single word–picture matching, sentence comprehension). Studies addressing only semantics or phonology (i.e., rather than both domains) were excluded due to potential confounding factors that may influence activation patterns (e.g., sample differences) between studies. Only studies utilizing MRI or intraoperative data were included due to the inherent spatial advantage of these methods over others (e.g., EEG). Positron emission tomography (PET) studies were excluded due to potential challenges in aggregating data from these studies with MRI studies, which use different spatial templates and atlases. Finally, any structural studies (e.g., voxel-based lesion symptom mapping, voxel-based morphometry) that did not include statistical relationships between brain and behavioral data were excluded due to limitations on interpretability.

Results from the database searches were exported to Mendeley, and duplicates were removed. The first author screened all titles and abstracts. Undergraduate student research assistants identified and recorded the mean age of participants in the articles that passed the title and abstract screening process. The first author then reviewed articles including participants with a mean age of 60 or greater for the remaining eligibility criteria. The senior authors (ELM and SMH) re-screened approximately 10% of titles and abstracts and re-reviewed approximately 10% of full-text articles for inter-rater reliability. Once final eligibility decisions were made on the full text of the articles reviewed, the following details from each included study were aggregated in a spreadsheet: (1) article citation; (2) atlas or parcellation used; (3) behavioral tasks used; (4) contrasts and analyses used; (5) regions associated with semantics only and coordinates, if provided; (6) regions associated with phonology only and coordinates, if provided; (7) regions associated with both semantics and phonology and coordinates, if provided; (8) multiple comparisons correction technique, if used; and (9) population (e.g., neurologically intact adults, post-stroke aphasia).

Imaging findings were presented in two ways. First, Figures were created that provided a visual representation of the collective findings, agnostic to the population (i.e., neurologically intact or clinical sample) and task type. These Figures were created by first compiling all regions implicated in semantics, phonology, or both domains by each study. Then, studies were grouped according to the atlas used to analyze and report their results in the original research article (e.g., Harvard–Oxford [49]). A mask was created for each atlas, containing the frequency counts for the number of studies implicating each atlas region in semantics and/or phonology. The totals for each atlas were then combined and projected onto the FreeSurfer [50] inflated template brain surface (using the fsaverage function) and smoothed to produce the final Figures. Therefore, the Figures reflect the totality of brain region information extracted from all studies included in the scoping review. To maximize interpretability of the Figures, the Harvard–Oxford atlas [49] and the XTRACT atlas [51] were used to define the borders of gray matter and white matter regions, respectively, within the Figures.

Second, the imaging findings are presented as count data reflecting the frequency with which regions of interest (ROIs) were implicated in semantics, phonology, or both domains within neurologically intact versus clinical subsamples and across all included studies. In this reporting of the results, ROIs were identified in two ways. First, in a data-driven approach, regions were classified as ROIs if they were implicated in semantics, phonology, or both domains in approximately one-fourth of the studies included in the scoping review. Acknowledging this threshold as arbitrary, our second, hypothesis-driven ROI identification method was to include regions cited as crucial for semantic and phonological processes within neurobiological models of language processing (see the Introduction), regardless of the frequency with which they were indicated in the studies included in the scoping review. Hypothesis-driven ROIs included the left superior and middle frontal gyri, inferior frontal gyrus (all three parts), precentral gyrus, planum temporale, superior temporal gyrus, middle temporal gyrus, supramarginal gyrus, and angular gyrus, as well as four left-hemisphere white matter association tracts (i.e., the arcuate, inferior longitudinal, inferior fronto-occipital, and uncinate fasciculi). Next, each ROI was classified as having the potential for semantic or phonological specialization. ROIs were deemed potentially specialized for a given domain if they were implicated in more than twice the number of studies for one domain as they were for the opposite domain or both domains (e.g., 10 studies implicating a region in semantics, with four each implicating the same region in phonology or both domains). Notably, because of the breadth of this scoping review, we did not distinguish between regions implicated in the representation of language and those that control access to language; therefore, our results may include regions involved in either capacity, which we address in the Discussion (Section 4.4).

Notably, if multiple analyses or experiments were run within a single study, we collapsed the results across all analyses within the study so that in the results (i.e., Figures and counts), each study could appear only once as identifying a relationship with a given brain region (rather than being listed twice if two separate analyses supported a relationship between a brain region and phonology, for example). We chose to do this while aggregating the results for two reasons: first, to simplify the results, and second, because many of the studies that met the inclusion criteria for the scoping review came out of the same laboratories and included overlapping participant samples. Collapsing results across all analyses within a single study prevented further potential inflation of the aggregated results.

## 3. Results

**Figure 1:**
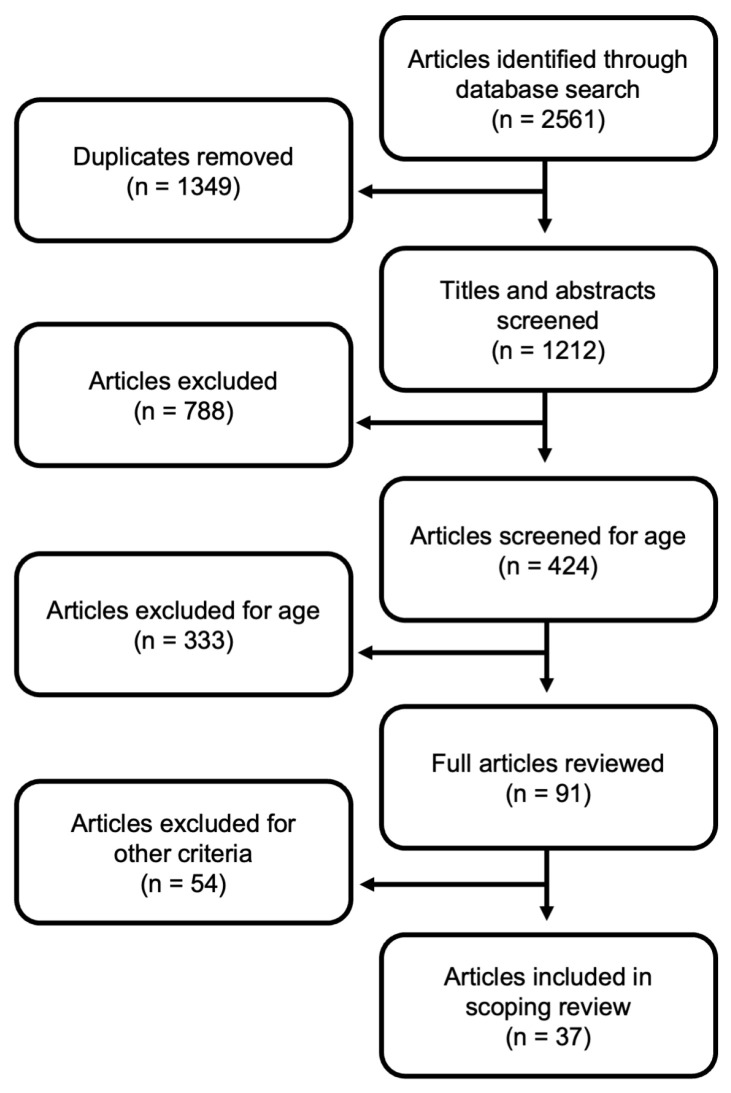
PRISMA diagram of screening and review processes.

The screening and review process, with the number of articles examined and excluded at each stage, is depicted in Figure 1, as recommended by the PRISMA extension guidelines [46]. The PRISMA extension guidelines checklist is also included as Supplementary Material, Table S1. The three database searches produced a total of 2561 results. Duplicates within each database were removed, yielding a total of 2093 results. Next, duplicates across databases were removed, yielding 1212 unique articles. Initially, 424 articles passed the title and abstract screening, but 333 were screened out for the mean age of participants. Of the 91 full-text articles reviewed by the first author for other eligibility criteria, 54 were excluded for methodological reasons. Twenty seven studies were excluded due to not statistically analyzing the relationship between brain and behavior (many of these were case studies), 14 were excluded due to the behavioral tasks (e.g., not including both a semantic and phonological task), eight were excluded for problems with the methods or reporting of regional localization (e.g., using angle of deep brain stimulator insertion instead of ROIs), three were excluded for age-related problems with the analysis (e.g., distinct age groups were collapsed), and two were PET studies. After these exclusions, 39 papers remained. Two of these papers [52,53] that met all other inclusion criteria were excluded because their study design and results could not be aggregated with the other included studies. Xing et al. [53] used probabilistic tractography to reconstruct white matter tracts between cortical nodes activated by a naming task. Their results consisted of relating phonological or nonverbal semantic processing with direct pathways between two regions (e.g., left anterior superior temporal gyrus to left orbital inferior frontal gyrus). This differed from all other studies included in the scoping review, which identified discrete cortical regions or white matter association tracts. Clark et al. [52] used large regions of interest in their gray matter volume analysis that did not allow for discretely localizing semantic and phonological skills to individual regions as in the other studies. Therefore, a total of 37 papers were included in the scoping review.

Inter-rater reliability for abstract screening was 90%. Inter-rater reliability for full-text article review was 80%. The first author met with the authors who screened and reviewed the articles for reliability to reconcile any discrepancies and reach consensus through discussion of the eligibility criteria and specific manuscript details. Although most abstracts and full-text articles were reviewed only by the first author, which may have impacted study selection, satisfactory reliability from 80 to 90% suggests that the studies selected for the scoping review would not have differed dramatically if completed by another author.

Publication years for the included articles ranged from 1999 to 2022. Fourteen studies were conducted in the United States, eleven in the United Kingdom, two each in Australia, Canada, and Germany, and one each in Finland, Norway, South Korea, Spain, Switzerland, and Taiwan. To answer the questions of interest, related to localizing the semantic and phonological networks in the brain, several different methodologies were employed by the included studies, and some studies utilized more than one approach (see Supplementary Tables S2 and S3). Most studies used either a lesion-based approach (*n* = 16; e.g., voxel-based lesion symptom mapping or voxel-based correlational methodology) or task-based functional MRI activation (*n* = 11). Additional methodological approaches included analysis of white matter measures (*n* = 2; e.g., diffusion-weighted imaging), gray matter density/volume (*n* = 7), and cortical thickness (*n* = 1).

### 3.1. Participants

The number of participants included in each study and the sample populations are shown in Table 2. There was a wide range of sample sizes, from 11 to 1231 participants in a single study, though most studies included under 100 participants. Due to the age criterion, the mean age was similar across studies, but ranged from 60 to 79 years, excluding mean ages for younger control samples that were used in comparison with older participants within the same study. Eight studies included only neurologically intact participants. Of these, six included both older and younger adult samples in order to directly compare the two groups. Out of 18 studies with a post-stroke population, 15 specifically included participants with post-stroke aphasia. Five studies included participants with primary progressive aphasia (PPA) or semantic dementia (equivalent to the semantic variant of PPA). Additional studies included participants with other neurodegenerative diagnoses (*n* = 5), including Alzheimer’s dementia (*n* = 2) and Parkinson’s disease (*n* = 2). One study specifically included participants with mild cognitive impairment (MCI).

### 3.2. Behavioral Tasks

The type of behavioral task(s) utilized in each study, also shown in Supplementary Tables S2 and S3, varied considerably, but trends were observed. The most common types of tasks, particularly for fMRI studies, were category (e.g., naming animals, fruits, and tools) and letter (i.e., naming words that start with a certain letter) fluency (*n* = 12 [30,54,55,56,57,58,59,60,61,62,63,64]). It was also common to utilize a custom neuropsychological battery of tests that included subtests of standardized assessments addressing semantics (e.g., synonym judgment, picture matching) and phonology (e.g., nonword discrimination, minimal pair discrimination), among other cognitive and linguistic domains (*n* = 11 [65,66,67,68,69,70,71,72,73,74]). Scores from these batteries were typically entered into a principal components analysis to identify unique factors representing particular cognitive or linguistic domains (e.g., semantics, phonology). Next, several studies utilized judgment or matching tasks (e.g., semantic or phonological decision, picture–word matching, and rhyming; *n* = 7 [75,76,77,78,79,80,81]), often during fMRI. Two studies each involved reading tasks (e.g., pseudowords vs. irregularly spelled words [82,83]) or analysis of error types during naming or description tasks (e.g., semantic vs. phonemic paraphasias [84]). Finally, there were three studies using less common tasks: one used a “Wisconsin Word Sorting Task,” sorting words by meaning, syllable onset, or rhyme [85]; another used a naming task with phonemic or semantic distractors [86]; and the last study involved treating distinct words with Semantic Feature Analysis vs. Phonological Components Analysis [87].

**Table 2:** Participant demographics.

Study	*N*	Age (Years)	Gender ^a^	Sample	Hand ^b^
Alyahya et al. (2018) [65]	48	63.31 (41–87)	14 F 34 M	Chronic post-stroke aphasia (12 m)	48 R
Alyahya et al. (2020A) [67]	46	63.21 (44–87)	14 F 32 M	Chronic post-stroke aphasia (12 m)	46 R
Alyahya et al. (2020B) [66]	42	63.11 (44–87)	14 F 28 M	Chronic post-stroke aphasia (12 m)	42 R
Baldo et al. (2006) [54]	48	62.9 (43–80; SD = 9.6)	14 F 34 M	Chronic post-stroke aphasia (9 m)	48 R
Biesbroek et al. (2021) [55]	1231	66.6 (21–94; SD = 11.6)	464 F 767 M	Acute stroke	1179 R 12 L 21 A
Boukrina et al. (2015) [75]	11	62.9 (46–83; SD = 8.7)	7 F 4 M	Subacute stroke (5 w)	11 R
Brambati et al. (2009) [82]	10 56	61.7 (+/−8)	36 F 30 M	NI controls Neurodegenerative disease	NR
Butler et al. (2014) [68]	19 31	68.21 (59–80; SD = 5.99)	8 F, 11 M 5 F, 26 M	NI controls ^c^ Chronic post-stroke aphasia (12 m)	19 R 31 R
Chang et al. (2020) [56]	36 24 26	71.11 (SD = 5.97) 70.12 (SD = 7.79) 74.15 (SD = 8.49)	22 F, 14 M 16 F, 8 M 12 F, 14 M	NI controls S-MCI M-MCI	36 R 24 R 26 R
Chouiter et al. (2016) [57]	191	62.2 (SD = 14.9)	71 F 120 M	Subacute stroke or tumor	191 R
Ellfolk et al. (2014) [58]	28 28	61.3 (SD = 7.2) 60.3 (SD = 8.1)	13 F, 15 M 14 F, 14 M	NI controls PD	NR
Froehlich et al. (2018) [76]	58 25	70.4 (63–79, SD = 3.4) 25 (21–35, SD = 3.67)	27 F, 31 M 18 F, 7 M	Older NI Younger NI ^c^	58 R 25 R
Halai et al. (2017) [69]	31	64.32 (45–84)	5 F, 26 M	Chronic post-stroke aphasia (12 m)	31 R
Halai et al. (2018) [70]	46	65.46 (SD = 11.49)	13 F, 33 M	Chronic post-stroke aphasia (12 m)	46 R
Henry et al. (2012) [71]	15 15	67.8 (SD = 8.5) 71.6 (SD = 7.7)	7 F, 8 M 6 F, 9 M	NI controls ^c^ PPA	14 R, 1 L 14 R, 1 L
Martins et al. (2014) [85]	14 14	63 (+/−8) 26 (+/−5)	6 F, 8 M 8 F, 6 M	Older NI Younger NI ^c^	14 R 14 R
Meinzer et al. (2012) [30]	14 14	69.2 (61–80, +/−5.8) 24.6 (19–32, +/−4.4)	7 F, 7 M 7 F, 7 M	Older NI Younger NI ^c^	14 R 14 R
Pereira et al. (2009) [59]	32	73.1 (SD = 5.9)	20 F, 12 M	PD	NR
Riello et al. (2022) [60]	35	67.74 (51–82, SD = 7.6)	16 F, 19 M	PPA	NR
Rizio et al. (2017) [86]	20 20	67.25 23.7	15 F, 5 M 10 F, 10 M	Older NI Younger NI ^c^	20 R 20 R
Rochon et al. (2010) [77]	10 4	61 67.25 (50–83)	3 F, 7 M 1 F, 3 M	NI controls Chronic post-stroke aphasia (12 m) ^c^	10 R 4 R
Rodriguez-Aranda et al. (2016) [61]	24 18	66.21 (SD = 8.96) 64.94 (SD = 9.57)	9 F, 15 M 9 F, 9 M	NI controls AD	NR
Saykin et al. (1999) [78]	6 9	71 (SD = 4) 79 (SD = 5)	4 F, 2 M 3 F, 6 M	NI controls AD ^c^	13 R 2 L
Schmidt et al. (2019) [62]	85	63.97 (22.4–85.8)	23 F, 62 M	Chronic post-stroke aphasia (5 m)	NR
Schumacher et al. (2019) [72]	38	64 (45–88)	11 F, 27 M	Chronic post-stroke aphasia (12 m)	38 R
Shafto et al. (2012) [79]	16 14	75.75 (SD = 4.99) 23.86 (SD = 4.14)	NR	Older NI Younger NI ^c^	NR
Sonty et al. (2003) [80]	11 11	66.5 (+/−6.7) 63.4 (+/−4.6)	6 F, 5 M 6 F, 5 M	NI controls PPA ^c^	11 R 11 R
Stark et al. (2019) [84]	57	61.68 (+/−12.02)	25 F, 32 M	Chronic post-stroke aphasia (6 m) ^d^	57 R
van Hees et al. (2014) [87]	14 8	61.71 (49–81, SD = 10.07) 56.38 (41–69, SD = 9.15)	8 F, 7 M 5 F, 3 M	NI controls Chronic post-stroke aphasia (12 m) ^c^	14 R 8 R
Vonk et al. (2019) [63]	505	74.1 (62–96)	281 F, 224 M	NI	461 R 32 L 11 A
Wilson et al. (2009) [83]	9 5	65.7 61.4	7 F, 2 M 4 F, 1 M	NI controls SD (PPA) ^c^	7 R, 2 L 4 R, 1 L
Wilson et al. (2010) [88]	10 60	68.5 (SD = 5.9) 65.85 ^e^	5 F, 5 M 34 F, 26 M	NI controls ^c^ PPA + dementia	9 R, 1 L 44 R, 6 L
Woollams et al. (2018) [73]	43	64.27 (44–87)	NR	Chronic post-stroke aphasia (12 m)	43 R
Zhang et al. (2013) [64]	344	78.3 (SD = 4.8, 70–90)	187 F, 157 M	NI	321 R, 11 L, 12 A
Zhao et al. (2018) [74]	35	63.8 (44–86)	12 F, 23 M	Chronic post-stroke aphasia (12 m)	35 R
Zhao et al. (2020) [89]	70	65.21 (44–87)	17 F, 53 M	Chronic post-stroke aphasia (12 m)	70 R
Zhuang et al. (2016) [81]	20 20	66.6 (60–78) 23.7 (19–34)	12 F, 8 M 10 F, 10 M	Older NI Younger NI ^c^	20 R 20 R

Note. ^a^ Gender was only reported as male or female in the included studies, and therefore, no other genders (e.g., non-binary) are reported here. ^b^ For post-stroke participants, pre-morbid handedness is reported. ^c^ These groups of participants were not analyzed for the main results of the scoping review, due to the mean age or the methods used (e.g., task-based fMRI activation in a clinical sample). ^d^ In the study by Stark et al. (2019) [84], only the Philadelphia Naming Test group was used, due to the mean age of the other group being below our minimum. ^e^ A weighted average was taken of the ages provided for all PPA and dementia groups. A = ambidextrous or other, AD = Alzheimer’s dementia, F = female, L = left, M = male, MCI = mild cognitive impairment, M-MCI = multi-domain MCI, NI = neurologically intact, NR = not reported, PD = Parkinson’s disease, PPA = Primary progressive aphasia, R = right, and S-MCI = single-domain MCI.

### 3.3. Imaging Results

**Figure 2:**
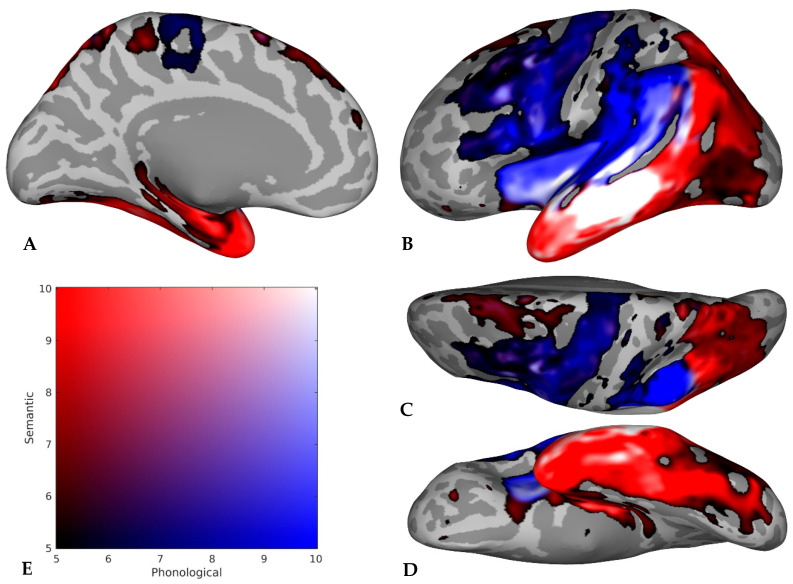
Semantic and phonological regions in older adults. Note: Medial (**A**), lateral (**B**), dorsal (**C**), and ventral (**D**) perspectives shown, as well as a legend for the number of studies linking a particular region to semantics and/or phonology (**E**). Data based on 3486 participants across 36 studies reporting older adult results. Where more than 10 studies implicated a region in semantics, phonology, or both, the coloring is the same as for 10 studies (i.e., red, blue, or white, respectively). Notably, the parcel labeled “middle temporal gyrus—posterior” in the Harvard–Oxford atlas includes territory that is centrally located within the middle temporal gyrus, likely contributing to the area of greatest overlap (in white) appearing more central, bordering on anterior middle temporal gyrus, rather than being further posterior.

**Figure 3:**
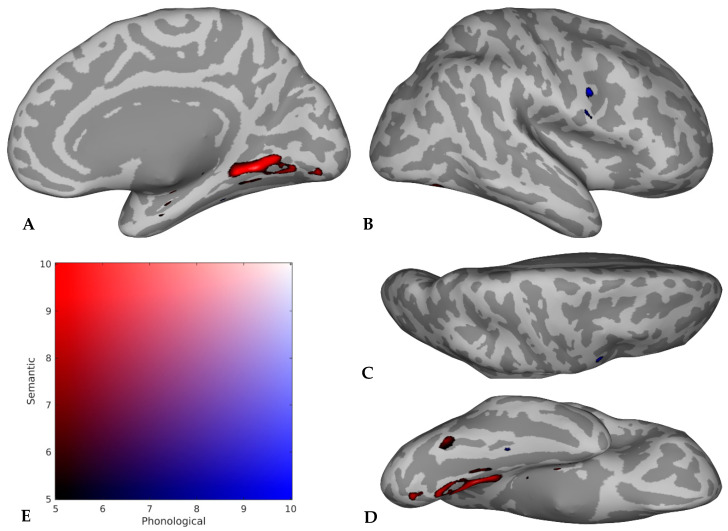
Semantic and phonological regions in older adults. Note: Medial (**A**), lateral (**B**), dorsal (**C**), and ventral (**D**) perspectives shown, as well as a legend for the number of studies linking a particular region to semantics and/or phonology (**E**). Data based on 3486 participants across 36 studies reporting older adult results. Where more than 10 studies implicated a region in semantics, phonology, or both, the coloring is the same as for 10 studies (i.e., red, blue, or white, respectively).

The primary results of the scoping review are depicted in Figure 2 and Figure 3, highlighting the distribution of semantics and phonology in the left and right hemispheres, respectively, of older adults. These Figures take advantage of the full dataset available from the scoping review to provide a collective picture, which is useful in light of the fact that we identified a relatively small number of studies with only neurologically intact older adults or only clinical samples, and four studies [56,58,61,82] included both types of participants in their analyses. However, one study [81] was not included in the development of the Figures because this study did not report results in their older participants alone. Instead, they reported their results as the contrast between older and younger participants. We did not want this contrast to influence the Figures; however, this study was still included in the scoping review to descriptively report their findings contrasting younger and older participants (see Section 3.4, below). Note that these Figures are not meta-analytic (e.g., based on activation likelihood estimation), nor are they weighted based on sample size or effect sizes. Rather, each Figure is a descriptive summary of the frequency with which these regions are implicated in the literature. Images were smoothed to improve visualization, but given the varying atlases and parcellation schemes used in the primary literature, caution is warranted in the interpretation of spatial precision.

Nonetheless, several meaningful conclusions can be drawn from these Figures. First, clear left-hemisphere lateralization is seen for both semantics and phonology, as evidenced by the large swathes of tissue implicated in Figure 2 compared to the sparseness of Figure 3. Indeed, 13 left cortical regions (i.e., the middle frontal gyrus [MFG]; inferior frontal gyrus-pars opercularis [IFGop]; superior, middle, and inferior temporal gyri [STG/MTG/ITG]; insula; temporal pole [TP]; Heschl’s gyrus [HG]; fusiform cortex [FC]; hippocampal/parahippocampal gyrus [H/PG]; supramarginal gyrus [SMG]; angular gyrus [AG]; and lateral occipital cortex [LOC]) were implicated in semantics, phonology, or both domains in 10 or more studies. In contrast, no regions within the right hemisphere were indicated in at least 10 studies, but the right lingual and precentral gyri were implicated in semantics and phonology, respectively, as shown in Figure 3. Second, the general spatial distribution of regions with possible semantic versus phonological specialization follows the proposed respective ventral/dorsal pathways, demonstrating that findings from older adults generally adhere to a dual-route architecture.

A more nuanced summary of the imaging results is included in Table 3 to provide interpretive guidance to the Figures. Study-level count data for neurologically intact and clinical samples are included in Supplementary Tables S2 and S3, respectively. Given that 37 studies were included in the scoping review, the benchmark of 10 studies was selected to define the data-driven ROIs for semantics and phonology (or both domains). Across all samples, several regions were implicated more often for semantics than phonology. Notably, though, several ROIs had either higher dual-domain than single-domain counts (e.g., MTG, FC, and LOC) or similar single-domain and dual-domain counts (e.g., IFGop, Ins, and ILF). Only the left arcuate fasciculus (AF) had counts for a single domain (i.e., phonology) with no studies implicating it in the other (i.e., semantics) or both domains. Other ROIs with greater phonology than semantic counts and low both-domain counts—indicating potential for phonological specialization—included the left precentral gyrus (PreG), HG, PT, and SMG. ROIs with greater semantic than phonology counts and low counts for both domains (i.e., those with the highest potential for semantic specialization) included ITG, TP, H/PG, FC, and uncinate fasciculus (UF). Regions with high counts for both domains and/or similar semantic and phonology counts are less likely to be specialized for either domain in older adults; these regions included most prefrontal ROIs (i.e., superior frontal gyrus [SFG], MFG, and all three parts of IFG), the insula, STG, MTG, inferior fronto-occipital fasciculus [IFOF], and inferior longitudinal fasciculus [ILF].

The regions implicated in semantics versus phonology generally aligned across neurologically intact versus clinical samples. However, the percentage of studies reporting findings for the included ROIs was generally higher for clinical versus healthy samples, possibly due to the high number of samples drawn from lesion-symptom-mapping studies involving participants with post-stroke aphasia. Brain damage in these individuals tends to be limited to regions supplied by the left middle cerebral artery territory, thereby restricting the search space in these studies to the left perisylvian cortex rather than the entire brain. The implications of these collective findings are addressed in greater detail in the Discussion.

### 3.4. Older and Younger Adult Comparisons

Six of the studies in our scoping review [30,76,79,81,85,86] included and directly compared older and younger participants. Among these six studies, it was common for older adults to demonstrate positive activity that exceeded that of younger adults for some of the targeted tasks, in a variety of brain regions [30,76,85,86]. It was less likely for younger adults to have positive activity exceeding that of older adults, but this did occur in some cases (e.g., when the comparison between younger and older adults consisted of contrasts reflective of greater demands, i.e., hard over easy, as in Shafto et al. [79]).

In the studies by Froehlich et al. [76] and Meinzer et al. [30], younger adults had no significant activity remaining when contrasted with that of older adults. Similarly, Rizio and colleagues [86] found no significant activity in younger adults, contrasted with older adults for their semantic condition (when contrasted with either their unrelated or phonological conditions). However, when the phonological was contrasted with the unrelated condition, they did find greater activity in the right postcentral gyrus, right supramarginal gyrus, and bilateral middle temporal gyrus in younger adults. When the phonological was contrasted with the semantic condition, they found greater activity in bilateral central opercular cortices, right insula, left putamen, bilateral precentral gyrus, bilateral postcentral gyrus, right supramarginal gyrus, right lingual gyrus, bilateral precuneus, and bilateral cuneus in the younger group. The opposite pattern was shown by Martins et al. [85]. When contrasting either of their two phonological conditions (rhyme and onset) with their semantic conditions, Martins and colleagues [85] found that younger adults had no remaining significant positive activity above and beyond that of older adults. However, when they contrasted their semantic condition with their onset condition, they found greater activity for younger adults in the occipital cortex (BA 17 and 18), and when contrasting their semantic condition with their rhyme condition, they found greater activity for younger adults in the ventrolateral prefrontal cortex (BA 47/12), posterior cingulate cortex (BA 23), inferior temporal cortex (BA 20), inferior parietal cortex (BA 40), precuneus (BA 7), and occipital cortex (BA 17).

In Shafto et al. [79], older adults had increased activity for low (compared to high) imageability words in the left middle/superior temporal gyrus. That said, a direct contrast did not reveal any regions with a stronger effect of imageability in older than younger adults, even though younger adults did not demonstrate any regions with a significant main effect of imageability. On the other hand, the younger group did demonstrate an effect of phonological cohort competition in the left inferior frontal gyrus, with greater activity when competition was higher. This effect was greater than in the older adults, who demonstrated no main effects of cohort competition. Younger adults also demonstrated a greater effect of imageability for high compared with low competition words in the left inferior frontal gyrus, bilateral cerebellum, and left supplementary motor area. Comparatively, older adults’ imageability effect did not differ based on cohort competition.

Finally, Zhuang et al. [81] found no significant age differences in their rhyme task, but did find differences in their semantic task. The semantic task elicited significantly greater activation in older than in younger adults in the left inferior frontal and fusiform gyri, as well as bilateral posterior cingulate. However, a significant positive correlation between left inferior frontal gyrus activity and performance on the semantic task was actually driven by the younger group. The authors suggest that the lack of a significant correlation among the older adults may reflect the limited power of their sample size (*n* = 20) or increased variability of behavior and brain activity in older adults, concluding that their results still support the notion of age-related preservation and enhancement of semantic abilities.

## 4. Discussion

The primary purpose of our scoping review was to explore the extent of semantic and phonological networks in older adults, based on the frequency of studies implicating a relationship with one domain, the other, or both domains. The results suggest some ROIs have the potential for semantic or phonological specialization in older adults, while other regions involved in both categories of tasks may be recruited due to a role in domain-general cognition or as sites of overlap between semantic and phonological networks. The secondary purpose of our scoping review was to compare the potentially specialized semantic and phonological ROIs in older adults from our results with the networks of younger adults. Due to only six studies in our scoping review conducting direct comparisons between older and younger age groups, we focus this part of the discussion on comparing our results to findings from the meta-analysis of the semantic and phonological networks in younger adults by Hodgson et al. [14].

### 4.1. Regions of Interest with the Potential for Semantic Specialization in Older Adults

Roughly one-fourth of studies in our scoping review implicated several left-hemisphere regions in semantics that were not as frequently implicated in phonology and have also been repeatedly implicated in semantics in extant literature (i.e., TP, ITG, FC, H/PG, and UF). More specifically, twelve studies implicated the left TP in semantics, with only two overlapping studies implicating the region in phonology (see the specific studies implicating these and subsequent regions in Supplementary Tables S2 and S3). An additional three studies found the left TP to be involved in phonology alone, though the group led by Woollams [73] implicated other anterior temporal regions in semantics. Overall, more studies with clinical, rather than neurologically intact, populations linked ITG with language performance at all (see Table 3). Six studies in our scoping review implicated the left ITG (or BA 20, without specifying a different structure, such as FC) in semantics, without indicating an anterior or posterior subdivision [59,60,62,71,73,76]. An additional four studies specifically reported the anterior subdivision of ITG [63,65,66,70], while six separately or additionally implicated the posterior subdivision [55,65,66,69,72,84]. Another study implicated BA 37 in semantics [54], which corresponds to the posterior temporal lobe, but whether the associated area was localized inferiorly is unclear. Meanwhile, only five studies in total implicated the left anterior [55,66], posterior [55,66,72], or unspecified ITG or sulcus [75,76] in phonology. While these results indicate some lack of consistency regarding subdivisions of the ITG, they are generally consistent with multiple models that prominently feature the anterior temporal lobe (encompassing the TP, as well as the anterior MTG and ITG) as a general amodal semantic hub [7,9] or a taxonomic semantic hub [8]. Notably, the posterior ITG appears in multiple models as well, as another convergence zone [6], a store of semantic knowledge [4,5], a multimodal conceptual processing region [7], and a component of the left frontoparietal network semantic control system [9].

Other cortical ROIs that fit our criteria for potential semantic specialization are FC and H/PG. A total of 17 studies suggested that FC plays a role in semantics, from the anterior temporal lobe to the occipital lobe. Specifically, six studies indicated the anterior portion [65,66,69,70,73,82] and five indicated the posterior temporal or occipital portion, including BA 37 [54,55,72,86,89], while six studies implicated the mid-fusiform or entire gyrus, without further localization [63,71,76,83,84,87]. These results align with the fusiform gyrus’s role as a convergence zone in multiple semantic models [6,7,9]. However, it should be noted that seven studies in our scoping review also implicated FC in phonology, including three highlighting the mid- or an unspecified part of FC [63,76,83] while four implicated the posterior or temporo-occipital FC [30,72,75,82]. As such, while the FC fits our semantic specialization definition, there may be less certainty with regard to portions of this cortex for semantics outside of potential convergence zones and well-known category-specific processing areas (e.g., for faces and shapes). Nine studies implicated either the left hippocampus [61,64,89] or neighboring PG [55,59,61,70,72,78] in semantics compared to only three studies implicating the left hippocampus [30,55,61] and/or left PG [61] in phonology. The H/PG are not typically singled out as unique components of semantic models but have been described as part of the distributed conceptual system, given their involvement in memory [6,7], as well as part of the default mode network, which plays a role in semantic processing [9].

Of the white matter tracts that served as ROIs in this review, only UF fell into the category of being potentially specialized for semantics based on our criteria. Importantly, our scoping review included fewer studies that examined lesions within white matter or used white matter analyses than those that emphasized gray matter lesions and analyses. Therefore, we considered the UF to be specialized for semantics, despite having evidence from fewer than 10 studies in support of this association. Specifically, six studies in our scoping review linked UF to semantic tasks; meanwhile, only two studies indicated that the left UF may play a role in phonology. In Friederici and Gierhan’s [3] model of the language network, UF is included as part of the ventral pathway, mapping sound onto meaning. The relative dearth of white matter analyses also contributed to relatively few studies implicating the ILF and IFOF, both of which have also been linked to connecting key semantic regions within the ventral pathway.

### 4.2. Regions of Interest with the Potential for Phonological Specialization in Older Adults

Left HG, PT, SMG, PreG, and AF were implicated in phonology based on the studies included in our review. Ten studies implicated either the left primary auditory cortex, HG, or BA 41 or 42 in phonology. As for semantics, only two studies reported left HG and BA 41/42 may be involved. Nine studies identified the left PT as being involved in phonology. Only one study suggested a role in semantics for the left PT, inclusive of Wernicke’s area. Thus, there is evidence that the neighboring HG and PT may be specifically implicated in phonology. As the primary auditory cortex, HG’s role in sound processing is established and likely the most important for phonological tasks that involve auditory stimuli. Our finding for PT is consistent with its featuring (insofar as it includes the region known as area Spt) in Hickok and Poeppel’s [2] dual-stream model as a sensorimotor interface, mapping phonological representations onto articulatory motor representations.

The left SMG was implicated in phonology by 16 studies in our scoping review: seven specifically implicated the posterior supramarginal gyrus [55,65,66,69,72,73,89] and two of these also implicated the anterior division [55,66], while the remaining nine did not specify a subdivision [30,54,57,70,71,74,75,82,85]. Meanwhile, only five studies found a role for the left supramarginal gyrus in semantics. These results are consistent with a central role of SMG in phonology, particularly in word production, phonetic encoding, articulation, word learning, or phonological working memory [10,11,12]. Although semantic models have proposed the SMG as one of several convergence zones [6,7,9], this role is not consistent with our findings [2,10,11,12,90].

Less clear are the implications for the final cortical region implicated in phonology: PreG. In our scoping review, the left PreG, or BA 4, was implicated in phonology by eight studies overall. Two studies also suggested the left-hemisphere PreG plays a role in semantics. There is less agreement among the discussed models for a domain-specific role of PreG in phonological processing. Instead, Indefrey [10] suggests that PreG is most likely involved solely in overt articulation.

The left AF is the only white matter ROI included in this scoping review with a high potential for phonological specialization, as it was reported for phonological tasks in seven included studies. Of note, the left AF was reported in fewer than 10 studies, but importantly, not a single study implicated the left AF in semantics. As noted by Friederici and Gierhan [3], the AF, which provides structural connections between regions within the dorsal stream, has historically been associated with repetition and disconnection syndrome [91] but has more recently been implicated in complex syntax [92,93]. These findings also do not preclude potentially different roles of separate segments of the left AF, as proposed by Catani and colleagues [94,95,96].

### 4.3. Regions of Interest Implicated in Both Semantics and Phonology in Older Adults

Somewhat surprisingly, more regions in Table 3 reflect ambiguity regarding their role in semantics and phonology than those that demonstrate a pattern of potential semantic or phonological specialization. There are three possible explanations for this result. First, these regions may truly mediate both semantic and phonological processes, although this may reflect a lack of spatial granularity due to some studies included in the scoping review not describing the precise location (in, e.g., MNI coordinates) or specific parcel (e.g., ventral angular gyrus, dorsal posterior middle temporal gyrus) of activated or lesioned tissue. Second, these regions may instead mediate the domain-general processes needed to complete the semantic and phonological tasks. Third, these regions may appear to play a role in both semantics and phonology for study-specific reasons, which could include the specific tasks utilized, the methods of analysis, or the specific participant samples.

Likely candidates for the first explanation—a lack of spatial granularity of the reported results—are the two regions cited most often in our review as playing a role in both semantics and phonology, i.e., the left STG and MTG. The left STG was implicated by 11 studies in semantics and 17 in phonology. The left MTG was implicated by 19 studies in semantics and 14 in phonology. Illustrating the issues with spatial specificity for MTG in particular, 10 studies broadly implicated the entirety of the gyrus or BA 21 in semantics compared to only four in phonology. A higher number of studies implicated anterior MTG in semantics (i.e., eight [65,66,68,69,70,73,82,89]) compared to phonology (i.e., four [55,66,73,89]), whereas a higher number of studies implicated posterior MTG in phonology (i.e., 11 [55,64,66,68,69,72,73,75,80,82,89]) compared to semantics (i.e., six [55,57,65,66,73,84]). However, some of these studies overlap, such that three reported both anterior and posterior MTG were involved in semantics [65,66,73] and four reported both anterior and posterior MTG were involved in phonology [55,66,73,89], convoluting the interpretation of which parts of MTG are crucial for semantic versus phonological processes. Patterns of connectivity between the frontal and temporal lobes from a cortico-cortical evoked potentials study [97] suggest that voxels implicated in semantics and phonology follow a respective anterior-to-posterior gradient within inferior frontal and temporal regions. However, other work has proposed that the posterior MTG is critical for semantic control [98,99], somewhat challenging these findings. Further clarifying the functional roles of subdivisions of these regions in older adults is an important future follow-up to this scoping review.

A lack of spatial granularity and consistency across included studies likely influenced our findings for other regions as well, particularly the LOC. The LOC was indicated in semantic processing in 10 studies from our scoping review. Five studies specifically implicated the superior lateral cortex [55,65,66,84,89], five (with some overlap) implicated the inferior lateral cortex [55,66,76,77,84], and three did not specify a subregion [63,83,86]. Despite its potential for semantic specialization, seven studies also suggested the LOC may play a role in phonology. Three of these specifically indicated the superior subregion [55,63,66], two indicated the inferior [55,76], and an additional three either did not specify a subregion [83,85] or implicated the middle occipital gyrus alone [77]. Based on these results, and as shown in Figure 2, it appears that the middle-to-superior components of the LOC may play a stronger role in semantics, while the inferior component may have more mixed involvement in semantics and phonology.

As mentioned previously, an anterior-to-posterior continuum of semantics to phonological processing has been suggested for the left inferior frontal cortex as well as temporal regions. However, support for this hypothesis was not found in our review. Surprisingly low frequency counts were indicated for two parts of IFG, IFGorb and IFGtri, and neither was identified as a region of potential specialization for either domain within our review. While higher frequency counts were found for left IFGop, this region was also not indicated as a site of domain-specific specialization. Without specifying a subregion, the IFG was found to be involved in semantics and phonology in one [61] and three studies in our scoping review [61,62,71], respectively. Only three studies reported left IFGorb solely for semantic tasks, whereas four studies implicated the region in phonology. Notably, an additional three studies implicated the more ventral and medial left orbitofrontal cortex in semantics [55,63,73], one of which also indicated its role in phonology [73]. Similarly, only a few studies included in our review implicated the left IFGtri in semantics or phonology. Moreover, five studies implicated IFGop in both semantics and phonology, three in phonology only, and one in semantics only. As such, these findings are in direct contrast to previous proposals of more anterior portions of IFG having a central role in semantic processing (such as goal-directed retrieval of semantic knowledge, semantic selection, and resolution of competition between candidate representations [6,7,16,100]) and posterior IFG having a crucial role in phonological encoding [10], articulation [2], and/or phonological unification processes [4,5]. However, our results—particularly with regard to posterior-dorsal IFG—are more consistent with the proposed spatial architecture of the multiple-demand system [101,102,103].

Other regions with a relatively similar split between domains include the left SFG, MFG, and AG. Our findings in the left SFG and MFG may be consistent with the role of the left dorsolateral prefrontal cortex in the control component of Hagoort’s MUC model [4,5], which involves relating language to action during processes such as conversational turn-taking or code-switching. Given the role of the left dorsolateral prefrontal cortex in verbal and spatial working memory [104] and the middle frontal gyrus, specifically in the multiple-demand network [20], these regions are likely to contribute to domain-general cognitive control mechanisms. Therefore, finding they have been linked with both semantic and phonological tasks in older adults may indicate engagement of more domain-general resources during language processing due to de-differentiation [36,37,105]. Alternatively, recruitment of SFG and MFG for both domains across a handful of studies may reflect the fact that commonly used tasks across several studies (e.g., category/letter fluency) required executive control in addition to language processes. Future work may test this question directly and determine, if present, whether such de-differentiation relates to task performance in a beneficial way, supporting a compensatory perspective (i.e., CRUNCH [31,32]) of age-related changes in brain activity.

The AG, on the other hand, has been specifically implicated in lexical and semantic feature representation [4,5] and, as part of the inferior parietal lobule, has been implicated in word production [10,11], word learning and phonological working memory [12], integrating sensory information [6,7,9], or acting as a thematic hub [8]. Hodgson et al. [14] identified a dissociation between dorsal and ventral AG, proposing that dorsal AG is involved in phonological and domain-general control, whereas they note considerable debate surrounding the role of ventral AG in semantic-specific representation vs. control mechanisms. The findings of Hodgson et al. [14] suggest that our results for the AG may be explained by the combination of missing granularity in localizing semantic and phonological skills within the AG, as well as its potential role in domain-general processes.

The insula’s being repeatedly implicated in both semantic and phonological skills by our scoping review may be due to task-specific patterns in the studies we included. Fourteen studies indicated left insular cortex involvement in phonology. The majority of these did not specify a particular portion of the insula [54,55,57,61,63,70,71,75,76,89], though three specified the posterior insula [68,69,73] and one specified the anterior portion [83]. About half as many studies suggested that the left insula was involved in semantics. This is a considerable number of studies, resulting in seven that implicated the insula’s involvement in both domains. However, the dual-stream model [2] includes the insula in the articulatory network of the dorsal stream. Meanwhile, Indefrey [10] highlights ongoing debate regarding the anterior insula’s role in articulatory planning versus execution. Given the insula’s potential role in articulation-related processes [2,10,90], it is possible that this result is due to the number of studies in our review that involved overt speech production tasks that taxed articulatory circuits (e.g., category and letter fluency). Thus, these results warrant further investigation to disentangle the role of the left insula in phonological versus articulatory processes.

As mentioned in our discussion of the UF, our scoping review revealed a relative dearth of studies examining white matter association tracts in older adults. In addition to the UF, this affected our findings for the ILF and IFOF, two additional tracts implicated as ventral pathways. For example, the ILF connects the LOC with the ventral/ventromedial anterior temporal lobe, suggesting a role particularly in transmitting visual information to the semantic system [7]. The IFOF connects the frontal cortex with the posterior temporal, occipital, and parietal cortex, with a proposed role in supporting semantic processes and comprehension [3]. In our scoping review, the left ILF was implicated in semantics by nine studies, but five of these studies also implicated the left ILF in phonology. The findings for the IFOF were similar. Eight studies implicated the left-hemisphere tract in semantics. Three of these and one additional study suggested the left IFOF was involved in phonology. A potential explanation for the lack of evidence in our scoping review supporting specialized roles in semantics for ILF and IFOF is that many of the studies reporting white matter findings were voxel-based correlational methodology studies that employed a principal components analysis on a comprehensive neuropsychological battery to identify semantic and phonological components. Perhaps these components, specifically the phonological component, included visual processing or comprehension elements that corresponded to ILF/IFOF integrity. However, the overall low frequency counts for ILF and IFOF made interpreting their findings from this scoping review challenging and highlights the need for future work, in which these pathways are interrogated for both semantic and phonological tasks.

### 4.4. The Potential Effects of Aging on Semantic and Phonological Networks

One of the purposes of our scoping review was to compare the semantic and phonological networks in younger and older adults. Our intention was to address observed differences within the context of cognitive aging models that each make specific predictions about age-related changes in neural activity during cognitive functioning. These models include HAROLD [26,27], which proposes reduced lateralization and should result in greater right-hemisphere involvement in language; CRUNCH [31,32], which proposes compensatory overactivation and could lead to recruitment of additional focused resources outside territory traditionally activated for a particular language tasks; de-differentiation [36,37], which similarly proposes reduced specialization and alternative recruitment of widespread, generalized resources; and PASA [33], which proposes reduced activity in posterior regions and increased activity in anterior prefrontal regions. We planned to do this by reviewing the results of studies in our scoping review that directly compared older and younger participant groups. However, there was a very limited number of studies that conducted such a direct comparison. Among the six studies in our review that directly compared older and younger adult samples, there was no consensus in terms of differences in the semantic and phonological brain networks. This is likely the result of vast differences in behavioral tasks, methods, and analyses. However, within this small number of studies, one common pattern was for older adults to demonstrate more activity than younger adults in shared brain regions associated with semantics or phonology, which could reflect an increase in effort or task-demand for the older groups [76], which is consistent with the CRUNCH [31,32] perspective or potentially the de-differentiation hypothesis. Notably, when younger adults were more likely to demonstrate greater activity, it was in the context of more cognitively demanding contrasts (e.g., hard > easy, linguistic task > control), which may reflect the greater difference in effort that younger adults exert for more challenging tasks than easier ones. Thus, our results may reflect age-related changes in cognitive effort such that easier tasks are commensurately challenging for older adults as intentionally challenging tasks are for younger individuals. Ultimately, more studies comparing neural activity associated with both semantics and phonology in older and younger adults are needed to draw firmer conclusions.

Due to the limited number of studies directly comparing older and younger adults, we also compared our results with the results of the meta-analysis in younger adults by Hodgson and colleagues [14]. Despite methodological differences between this review and that of Hodgson et al. [14] (e.g., review type, investigation into representation vs. control processes), there were similarities between our findings. Similar to our results regarding ROIs with the potential for semantic specialization, Hodgson et al. [14] found support for the anterior temporal lobe (including the TP) and PG playing a greater role in semantics than phonology. The authors endorse the anterior temporal lobe as the core of semantic representation. They also support the notion that phonological representation is primarily isolated to the posterior STG, which is consistent with its role in neurobiological models of phonology [2,4,5,10,11,12]. Although we found a large number of studies in our scoping review that reported the STG to be involved in both semantics and phonology, not all included studies that localized their findings within the STG, contributing to a lack of granularity. This is supported by the posterior STG position of Hodgson and colleagues’ [14] phonological representation ROI and other work implicating the anterior STG specifically in semantics [106]. Moreover, more studies in our scoping review linked HG and PT with phonology rather than semantics, both of which are in close proximity to and potentially overlap with posterior STG. Finally, similar to our finding of potential phonological specialization for the SMG, Hodgson et al. [14] also found the SMG to be more active for phonological compared to semantic tasks. Despite this finding, the authors interestingly propose that the SMG’s role is one of domain-general cognitive control due to its presence in the results of a separate working memory meta-analysis and the multiple-demand network [20].

Hodgson and colleagues [14] propose that additional regions implicated in semantics and phonology are in fact recruited due to their role in domain-general control mechanisms, rather than being truly specialized for language. These regions include the posterior ITG, whereas we identified the ITG as potentially specialized for semantics. Although this difference at the ITG—finding the potential for semantic specialization in older adults compared with less specialization in younger adults—may appear to contradict the de-differentiation hypothesis, our finding may again be attributable to a lack of granularity, due to several studies in our scoping review not specifying an anterior vs. posterior subdivision. However, as noted in Section 4.1, more of our included studies implicated the posterior rather than the anterior division in semantics, and the posterior division has specifically been implicated in semantic models [4,5,6,7,9]. Hodgson et al. [14] propose a similar domain-general role for left dorsolateral prefrontal cortex in their model of the language network, which includes SFG and MFG, and is consistent with our findings suggesting involvement in both semantics and phonology for these two regions (although, by fewer than 10 studies).

Contrary to our findings, Hodgson’s group [14] indicated a role for IFGorb, posterior MTG, and ventral AG in semantic control (though they note debate regarding AG’s role in semantic representation vs. control). As discussed in Section 4.3, a lack of granularity in spatial localization may have contributed to our lack of finding potential semantic specialization in the posterior MTG and ventral AG in our scoping review. However, the same may not be the case for IFGorb. Apart from the three studies in our scoping review that linked the activity or integrity of IFGorb, specifically, with semantic skills, only one other study implicated the IFG more broadly (without specifying a subdivision) in semantics [61]. An alternative possibility is that the studies in our scoping review did not capture IFGorb’s specific role in semantic control if, for example, this region is activated in more cognitively demanding tasks that were not well represented in our included studies. This may be surprising, considering the number of studies that utilized category/letter fluency tasks or neuropsychological batteries that included cognitive assessments. However, more of our findings came from clinical than neurologically intact samples, which means other tasks utilized with the clinical samples may have skewed toward simpler, less challenging semantic and phonological tasks. This is probably the most likely explanation for our findings, particularly in light of recent work demonstrating overactivation of prefrontal regions in older adults with greater task demands [107]. Although our findings at IFGorb are inconsistent with the PASA hypothesis, perhaps directly addressing the issue of representation vs. control by incorporating tasks with sufficient cognitive demand could alter outcomes in future work.

In our scoping review, the FC met the criteria for semantic specialization. However, this region does not feature prominently as part of the models proposed by Hodgson et al. [14]. The group led by Hodgson [14] did report the FC as part of their peak at the PG in the semantic > phonology contrast, but also found a small peak at the FC for the phonology > semantic contrast. Perhaps the lack of granularity in our scoping review did not capture the unique contributions of different components of the FC to semantics and phonology, particularly outside well-known category-specific processing areas (for e.g., faces or shapes). However, given meta-analyses implicating the FC in higher-order visual recognition processes such as face recognition [108,109], visual imagery [110,111], and reading [112,113], it is reasonable to find that it may be part of a specialized semantic network in our scoping review.

One difference that we anticipated was a potentially larger number of right-hemisphere regions being involved in semantics, phonology, or both for older adults, based on accounts of de-lateralization in the prefrontal cortex and language network that accompanies aging, according to the HAROLD model [26,27,42]. However, we did not see prominent involvement of the right hemisphere in the present scoping review. Hodgson et al. [14] identified several right-hemisphere regions in the activation likelihood estimation analyses for semantics and phonology, which largely overlap, most notably, at the right inferior frontal gyrus and dorsomedial prefrontal cortex. On the other hand, they did not identify any right-hemisphere regions uniquely contributing to either language domain in their formal contrasts. Overall, our results do not reflect a pattern consistent with the HAROLD model in the language network of older adults. However, this was likely influenced by our methodological choice to include lesion studies, which are largely confined to the left hemisphere.

### 4.5. The Nature of the Evidence

As part of conducting our scoping review, we explored the nature of the evidence examining semantic and phonological brain networks in older adults. Importantly, we narrowed our search to studies utilizing functional or structural MRI or cortical stimulation (i.e., during awake surgery) methodologies, thus eliminating studies utilizing EEG, PET, or other potentially informative techniques. Notably, there were few cortical stimulation studies included in our search results, and none met eligibility criteria after screening, typically due to a lack of clarity in the description of the behavioral tasks or a lack of statistical analysis. Within the MRI literature that met the inclusion criteria, we found a variety of methodologies used, including primarily lesion-symptom-mapping studies in post-stroke aphasia, gray matter volume mapping for PPA, and task-based fMRI in neurologically intact older adults. Among these, there was further variability in the analyses used, which dictated the extent to which we could draw conclusions related to the degree of distinction and overlap between the semantic and phonological networks. Some studies specifically completed conjunction or subtraction analyses that provide higher quality information, respectively addressing which brain regions are involved in both semantic and phonological tasks and which regions may be involved in one task more than the other.

Regarding the behavioral tasks utilized within the studies included in our review, there were a surprising number of investigations that incorporated the same task or methodological approach to data reduction. The most common fMRI task was category and letter fluency. Within lesion-symptom-mapping studies, several researchers used a principal components analysis on a custom neuropsychological battery (which typically resulted in at least one semantic and one phonological factor). Taken together, these two approaches accounted for roughly two-thirds of the studies in the scoping review. Although the neuropsychological battery used in the latter approach included a variety of semantic and phonological tasks, the same battery was used by most of the studies implementing this approach with little variation [65,66,67,68,69,70,72,73,74,89]. Implications of the lack of diversity among behavioral tasks included in the scoping review and the overrepresentation of these approaches are discussed further in Limitations.

Finally, we included studies analyzing data from either neurologically intact older adults or older adults with clinical conditions (e.g., post-stroke aphasia) due to the complementary evidence they provide. Due to the potential effects that reorganized and/or vulnerable networks from clinical samples may have on the results, we reported results separately, for each group, as well as combined in Table 3 to reveal potential differences. First, it is important to note that more of the studies utilized clinical, rather than neurologically intact, participants in the analyses relevant to our scoping review. However, even in proportional terms, the clinical studies appear to drive our results in most of the regions found to be specialized for semantics or phonology, especially in the ITG, TP, FC, and SMG, and in the STG and MTG, which appeared to be involved in both domains. For each of these regions, the clinical results alone would have met our threshold. This is an important finding that underscores the importance of considering evidence from clinical populations in future work. Both clinical and neurologically intact populations have historically informed neurocognitive language models and contributed different insights to the literature. As a result, incorporating both populations in meta-analytic examinations of semantic and phonological networks will provide an opportunity to probe these potential differences further, as well as cross-validate any comparable findings to yield more robust conclusions. However, limitations of studying clinical populations, particularly those with post-stroke lesions, are discussed further below.

### 4.6. Future Directions

A stated purpose of our scoping review was to gauge the potential for conducting a systematic review or meta-analysis addressing the same topic. Our results demonstrate that there is a large accumulation of evidence addressing the semantic and phonological neural correlates in older adults, which could indeed be used to conduct a review using one of these methods. Such a project would provide a compelling complement to existing work addressing this topic in healthy, younger adults [14]. Moreover, a meta-analysis would yield stronger, more reliable conclusions related to the topic than what is feasible with a scoping review. However, given the variety of methods, tasks, and populations included in the literature, it will be important to establish eligibility criteria to reduce confounds and maximize the interpretability of the results.

The activation likelihood estimation meta-analyses conducted by Hodgson et al. [14] did not include participants from clinical populations, only neurologically intact younger adults. Based on the results of our review, 17 of our included studies utilized a neurologically intact sample (either as the primary sample or a control for a clinical sample). However, only 11 of these included task-based fMRI activation methods, which may not be a sufficient number to conduct a meta-analysis. As a result, it may be worth adding studies targeting only semantics or phonology in an activation-based meta-analysis. In the present review, we excluded such studies in favor of those that examined both domains in the same sample. However, the meta-analyses conducted by Hodgson et al. [14] did include activation studies that only examined one domain or the other. Therefore, this could be an option to increase the sample size in a meta-analysis specifically targeting older adults. Additionally, Hodgson et al. [14] included PET studies, which is another option to improve the robustness of a potential meta-analysis addressing older adults. As a complement to an activation-based meta-analysis in neurologically intact older adults, we also recommend a lesion-based approach (i.e., anatomical likelihood estimation) to contribute regions critical for semantic and phonological processing. This combined approach is especially feasible in our target population of older adults, given the age at which aphasia and other acquired language disorders often occur, and would potentially provide strong cross-validation of activation-based results.

Finally, it was outside the scope of the present review to statistically analyze the relationship between regions implicated in semantics and phonology, such as with formal contrasts, but a future meta-analysis could fill this gap. Although a given region may be involved in both semantics and phonology, it may have a stronger role in or preference for one language domain over the other. In the present scoping review, we speculated as to the preference of certain regions for one domain over the other based on the number of studies that suggested a given region’s involvement in each language domain. However, a meta-analysis could determine statistical differences between each region’s involvement in semantics or phonology through conducting formal contrasts, as in Hodgson et al. [14].

### 4.7. Limitations

The present scoping review was limited by several factors. First among them is the issue of repeated participants. There were overlapping groups of participants in some study samples that were explicitly reported [68,69]. Additional participant samples likely overlapped in other studies that did not explicitly report it due to work being conducted within the same laboratories or recruiting from the same geographical area. Therefore, results from these studies may have effectively weighted the results of our scoping review in favor of findings that may be unique to the overlapping sample of participants included, rather than reflecting generalizable trends in the broader population. Conversely, our attempt to reduce the impact of any one study sample by collapsing results from each study across multiple analyses (if more than one analysis was presented) may have obscured potentially meaningful distinctions in the data.

Additional limitations of our scoping review primarily concern the different methodologies used by the included studies. For example, a main objective of our scoping review was to determine the extent to which semantic and phonological networks in older adults are distinct or overlapping. However, not all studies formally contrasted semantic and phonological tasks in order to statistically determine areas uniquely contributing to each function. Moreover, not all studies used methods to combine semantic and phonological outcomes (e.g., conjunction analysis) to determine areas contributing to both functions.

The presence of lesions in studies with post-stroke participants also contributed to methodological differences. For one, lesion size was inconsistently controlled across the included studies. Notably, Alyahya et al. [65] report different findings for regions associated with their semantic factor before and after controlling for lesion size, suggesting that this inconsistency may have contributed to differences in outcomes across studies. Another concern related to lesion studies is that these studies were limited in their ability to demonstrate hemispheric differences in older adults. Lesion-symptom-mapping studies of patients with damage isolated to the left hemisphere (e.g., due to stroke) could only reveal left-hemisphere brain regions associated with semantic and phonological skills. Out of the 37 studies included in the review, 16 reported lesion–behavior relationships [54,55,57,62,65,66,67,68,69,70,72,73,74,75,84,89], and 10 of these included participants with lesions only in the left hemisphere. Of the remaining six studies, four did not explicitly exclude participants with right-hemisphere lesions, but the presence of aphasia was an inclusion requirement, effectively limiting their sample to primarily participants with left-hemisphere lesions [69,70,73,74]. The other two studies report including participants with both left- and right-hemisphere lesions [55,57]. Left-hemisphere lesion-symptom-mapping studies were included in the review because we felt they could provide important insight as to the shared or specialized nature of the semantic and phonological networks in older adults, as well as the nature of the left hemisphere’s changing role in language as a result of aging. However, the absence of right-hemisphere involvement in these studies remains a limitation.

A broader limitation relates to the general inclusion of studies with clinical populations in this review, as opposed to analyzing only studies conducted on neurologically intact older adults. Our goal is to better understand the semantic and phonological networks in non-brain-injured older adults as a foundation for research examining the neural correlates of aphasia and its recovery. Given that part of the purpose of the scoping review was to gauge the nature of the evidence and the types of research studies that could begin to answer our questions, we chose to include samples of participants with neurological damage (e.g., post-stroke aphasia, Alzheimer’s dementia). Additionally, lesion studies provide important complementary evidence to functional neuroimaging studies [48], particularly because such studies allow for causal inference, unlike activation studies. We excluded studies examining fMRI activations in clinical populations to prevent the influence of their lesion, atrophy, reorganization, and/or recovery from impacting our exploration of intact semantic and phonological networks in older adults. However, including studies with clinical populations is not without problems. Most studies examining language in clinical populations cannot take into account any recovery that may have taken place when considering the relationship between the affected site (e.g., stroke lesion or location of reduced gray matter density in PPA) and behavior, unless they collected and included data from the acute phase or onset in their analysis, which is often not the case. Particularly in participants with chronic post-stroke aphasia, reorganization and recovery during the time since their stroke may have led to improvements in behavioral performance, resulting in differences between regions implicated in lingering deficits in PWA and regions necessary for those processes in neurologically healthy older adults. In other words, a damaged region may not appear to be as associated with a given function if the behavioral performance is not as severely impaired as it was shortly after the cerebrovascular accident.

There were additional limitations regarding our ability to identify differences between the language networks of older and younger adults. Many studies in this scoping review only included older adults, and we included these to address our primary question related to characterizing the semantic and phonological brain networks in this population. Because many studies did not provide an age range for their participants, we used the mean age for study eligibility and chose the age of 60 years as a minimum for these samples of older adults based on prior work using this mean age [43]. Although we suspect the influence is small, samples from some of the studies in our scoping review are known to include younger adults (and those without listed ranges may also include younger adults), despite having a mean age of 60 years or greater. Additionally, we were not able to take into account education, sociolinguistic variability, or cohort effects in our scoping review, despite recognizing that these are all important variables that may influence outcomes, particularly in cross-sectional aging research.

Some studies explicitly comparing older and younger adults’ brain activity related this differential activity in older adults to their behavioral performance. In such cases, this is helpful for determining whether differential activity is perhaps a maladaptive part of the aging process or compensatory in nature. For example, Meinzer et al. [30] found that activity in some areas was negatively correlated with accuracy on their in-scanner semantic task (i.e., postcentral gyrus/precuneus BA 3/7; medial/middle frontal gyrus, BA 6 and 9; middle/inferior frontal gyrus, BA 11), suggesting maladaptation. Similarly, Riello and colleagues [60] found that greater superior temporal gyrus volumes were associated with poorer letter fluency performance. However, many studies did not report such analyses exploring the relationship between brain activation and behavioral accuracy. As a result, it is not possible to disentangle which components of the semantic and phonological networks of older adults may facilitate linguistic abilities and which may contribute to their decline. Moreover, the study by Meinzer and colleagues [30] also reported differential negative activity in older adults (compared with the younger group) and its relationship to behavioral performance, which most research groups did not explore. They found that *more negative* activity in the postcentral/inferior parietal gyrus (BA 3/40) and the precuneus/cingulate gyrus (BA 7/31) was related to *better* performance on the in-scanner semantic task. Notably, researchers explicitly contrasted older and younger participant brain activations in only six of the studies included in our scoping review. The results of these studies provide unique insights resulting from the direct comparison of brain regions involved in particular tasks, but make up a small minority of the studies in the scoping review. Although we also compared our overall findings with the results of Hodgson et al. [14] in younger adults, a greater number of studies directly comparing older and younger samples would be beneficial.

Finally, we consider the types of behavioral tasks used in the included studies. Many of the included studies utilized verbal fluency tasks, which are commonly recognized to require not only semantic or phonological linguistic ability but also other cognitive elements, such as executive functioning, attention, initiation, and processing speed [114]. Moreover, semantic processing is involved in both semantic and phonemic fluency tasks due to the use of real words with meaning and the organization of word-retrieval processes in which semantically related items are more likely to be activated [115]. The same could be said for phonology influencing both tasks, given that phonological encoding is necessary to produce a given target word. Therefore, regions commonly activated by these two tasks may not reflect an overlap in semantic and phonological processing so much as an overlap in common executive processes or the semantics or phonology inherent in verbal fluency tasks of any kind. Other task concerns include the use of data reduction methods (e.g., principal components analysis) to isolate semantic and phonological processes [65,66,67,68,69,70,71,72,73,74,89]. Although these methods do successfully produce participant scores related to their performance on all tasks loading onto a given component, the resulting components are limited by the assessments entered into the analysis. If these assessments are not balanced in terms of task demands outside of semantics and phonology (e.g., working memory, attention, and inhibition), the resulting factors may be imbalanced as well (e.g., a phonological component may also be based on working memory ability, while a semantic component may also be based on inhibition). This is especially relevant considering the same battery of assessments, with little variation, was used in most studies employing this approach. However, there are also inherent limitations in the ability to create completely balanced tasks used to isolate semantic and phonological abilities.

Task difficulty also impacts performance and activation. Tasks that do not sufficiently challenge a participant may not yield expected activations, for example, in the right hemisphere of older adults [81]. Alternatively, tasks that are too challenging may also preclude expected activations. One solution to this problem of task difficulty is to use adaptive paradigms, such as those developed by Wilson et al. [116,117,118,119]. These studies were not included in the present scoping review due to the fact that the semantic and phonological tasks were not conducted within the same sample of participants, but the paradigms provide promise for improving our understanding of the degree of specialization and overlap within the semantic and phonological systems.

## 5. Conclusions

The present scoping review explored the semantic and phonological networks in the brains of older adults, the degree to which they were unique versus overlapping, and how they compared to the same networks in younger adults. We found evidence to support some brain regions and association tracts being potentially specialized for semantics (i.e., the left TP, ITG, FC, H/PG, and UF) or phonology (i.e., the left SMG, HG, PT, PreG, and AF), as well as some regions likely subserving both domains or other cognitive functions (i.e., the left SFG, MFG, IFG, STG, MTG, AG, ILF, and IFOF). Our results suggest subtle differences in the overlap of these networks in older compared with younger adults, which are generally compatible with the CRUNCH or de-differentiation perspective. However, it was outside the scope of this review to draw firm conclusions supporting or rejecting models of cognitive aging. We did not see a pattern in our results that was consistent with the HAROLD or PASA models. Our findings support the potential for future meta-analyses addressing the topic, particularly by combining activation and lesion-based approaches and including studies that investigated only semantics or phonology to make the best use of the available data. Such future work has the potential to improve our understanding of the semantic and phonological networks in older adults and, in turn, inform age-sensitive clinical applications, including cognitive screening and aphasia rehabilitation.

## Figures and Tables

**Table 1:** Search strategy for PubMed, Web of Science, and EBSCOhost databases.

Database	Terms	Refined by
Pubmed	((language[Title/Abstract] OR linguistic*[Title/Abstract] OR “language”[MeSH Terms]) AND (semantic*[Title/Abstract] OR “semantics”[MeSH Terms]) AND (phon*[Title/Abstract]) AND (mri[Title/Abstract] OR magnetic resonance imaging[Title/Abstract] OR neuroimaging[Title/Abstract] OR brain imaging[Title/Abstract] OR “neuroimaging”[MeSH Terms]))	language: English
Web of Science	TS=(language OR linguistic*) AND TS=(semantic*) AND TS=(phon*) AND TS=(mri OR magnetic resonance imaging OR neuroimaging OR brain imaging)	language: English
EBSCOhost	(language OR linguistic*) AND semantic* AND phon* AND (mri OR magnetic resonance imaging OR neuroimaging OR brain imaging)	language: English

**Table 3:** Summary of ROI results, by population sample and across all samples.

ROI	Neurologically Intact Samples (*N* = 1291 ^a^)			Clinical Samples (*N* = 2445 ^a^)			All Samples (*N* = 3486 ^b^)		
	Semantics	Phonology	Both ^c^	Semantics	Phonology	Both ^c^	Semantics	Phonology	Both ^c^
SFG	2 (12%)	0 (0%)	3 (18%)	1 (4%)	1 (4%)	1 (4%)	2 * (5%)	1 (3%)	4 (11%)
MFG	2 (12%)	2 (12%)	3 (18%)	0 (0%)	4 (17%)	1 (4%)	2 (5%)	5 * (14%)	4 (11%)
IFGorb	2 (12%)	2 (12%)	1 (6%)	0 (0%)	1 (4%)	0 (0%)	2 (5%)	3 (8%)	1 (3%)
IFGtri	1 (6%)	3 (18%)	2 (12%)	1 (4%)	1 (4%)	1 (4%)	2 (5%)	3 * (8%)	3 (8%)
IFGop	1 (6%)	1 (6%)	3 (18%)	0 (0%)	2 (8%)	2 (8%)	1 (3%)	3 (8%)	5 (14%)
PreG	0 (0%)	2 (12%)	1 (6%)	0 (0%)	5 (21%)	1 (4%)	0 (0%)	6 * (16%)	2 (5%)
Ins	1 (6%)	1 (6%)	3 (18%)	0 (0%)	6 (25%)	5 (21%)	1 (3%)	7 (19%)	7 * (19%)
STG	2 (12%)	2 (12%)	4 (24%)	2 (8%)	8 (33%)	4 (17%)	4 (11%)	10 (27%)	7 * (19%)
MTG	3 (18%)	1 (6%)	2 (12%)	5 (21%)	2 (8%)	10 (42%)	8 (22%)	3 (8%)	11 * (30%)
ITG	1 (6%)	0 (0%)	1 (6%)	9 (38%)	1 (4%)	3 (13%)	10 (27%)	1 (3%)	4 (11%)
TP	3 (18%)	1 (6%)	0 (0%)	9 (38%)	2 (8%)	2 (8%)	10 * (27%)	3 (8%)	2 (5%)
HG	0 (0%)	1 (6%)	0 (0%)	1 (4%)	8 (33%)	1 (4%)	1 (3%)	9 (24%)	1 (3%)
PT	0 (0%)	0 (0%)	0 (0%)	0 (0%)	8 (33%)	1 (4%)	0 (0%)	8 (22%)	1 (3%)
H/PG	2 (12%)	1 (6%)	1 (6%)	5 (21%)	0 (0%)	2 (8%)	7 (19%)	1 (3%)	2 * (5%)
FC	2 (12%)	1 (6%)	4 (24%)	10 (42%)	1 (4%)	2 (8%)	12 (32%)	2 (5%)	5 * (14%)
SMG	2 (12%)	3 (18%)	0 (0%)	1 (4%)	11 (46%)	3 (13%)	2 * (5%)	13 * (35%)	3 (8%)
AG	1 (6%)	1 (6%)	0 (0%)	2 (8%)	6 (25%)	3 (13%)	3 (8%)	6 * (16%)	3 (8%)
LOC	1 (6%)	1 (6%)	4 (24%)	3 (13%)	0 (0%)	2 (8%)	4 (11%)	1 (3%)	6 (16%)
AF	0 (0%)	0 (0%)	0 (0%)	0 (0%)	7 (29%)	0 (0%)	0 (0%)	7 (19%)	0 (0%)
ILF	0 (0%)	0 (0%)	1 (6%)	4 (17%)	0 (0%)	5 (21%)	4 (11%)	0 (0%)	5 * (14%)
IFOF	0 (0%)	0 (0%)	2 (12%)	5 (21%)	1 (4%)	3 (13%)	5 (14%)	1 (3%)	3 * (8%)
UF	0 (0%)	0 (0%)	1 (6%)	5 (21%)	1 (4%)	1 (4%)	5 (14%)	1 (3%)	1 * (3%)

Note: All regions are in the left hemisphere. A total of 17 studies included a neurologically intact sample, and 24 studies included a clinical sample. Of these, four studies included both. Regions implicated by more studies in semantics are shown in light red; phonology, in light blue. Regions frequently implicated in both domains maintain a white background. ^a^ Sample size includes some overlap in participant populations (i.e., neurologically intact older adults and clinical participants) for several studies because both groups were included in the analyses; see Supplementary Tables S2 and S3 for more details. ^b^ Studies that combined neurologically intact and clinical samples within analyses were counted once in the All Samples column. ^c^ Counts and percentages listed in the Overlap columns represent unique studies not already represented in the preceding Semantics and Phonology columns. * These counts include studies with combined neurologically intact and clinical samples; therefore, they do not equal the sum from the neurologically intact samples and clinical samples columns. AF = arcuate fasciculus, AG = angular gyrus, FC = fusiform cortex, HG = Heschl’s gyrus, H/PG = hippocampus/parahippocampal gyrus, IFGop = inferior frontal gyrus-pars opercularis, IFGorb = inferior frontal gyrus-pars orbitalis, IFGtri = inferior frontal gyrus-pars triangularis, IFOF = inferior fronto-occipital fasciculus, Ins = insula, ILF = inferior longitudinal fasciculus, ITG = inferior temporal gyrus, LOC = lateral occipital cortex, MFG = middle frontal gyrus, MTG = middle temporal gyrus, PreG = precentral gyrus, PT = planum temporale, SFG = superior frontal gyrus, SMG = supramarginal gyrus, STG = superior temporal gyrus, TP = temporal pole, and UF = uncinate fasciculus.

## Data Availability

The raw data supporting the conclusions of this article will be made available by the authors on request.

## Supplementary Materials

The following supporting information can be downloaded at: https://www.mdpi.com/article/10.3390/brainsci16030252/s1; Table S1: Preferred Reporting Items for Systematic reviews and Meta-Analyses extension for Scoping Reviews (PRISMA-ScR) Checklist. Table S2: Region of interest results from studies involving neurologically intact samples. Table S3: Region of interest results from studies involving clinical samples.
