# Supplementary material for: Semantic and Phonological Brain Networks in Older Adults: A Systematic Scoping Review
Source: Brain Sci. 2026 Feb 25;16(3):252. doi: 10.3390/brainsci16030252 (PMC13024727; doi:10.3390/brainsci16030252)
Supplement: Supplementary file 1 [file brainsci-16-00252-s001.zip › brainsci-4138054-supplementary/TableS2_NI.pdf]

Supplemental Table S2. Region of interest results from studies involving neurologically intact samples

Study	Method	Task	*N*	SFG	MFG	IFG orb	IFG tri	IFG op	PreG	Ins	STG	MTG	ITG	TP	HG	PT	H/PG	FC	SMG	AG	LOC	AF	ILF	IFOF	UF
Brambati et al. (2009) [82]	VBM	Reading irregular & pseudowords	66[a]								B	B		S				B	P	P					
Chang et al. (2020) [56]	DSI	Category & phonemic fluency	86[a]																					B	B
Ellfolk et al. (2014) [58]	VBM	Category & letter fluency	56[b]																						
Froehlich et al. (2018) [76]	fMRI	Semantic & phonological decision	58	B	B	B	B	B	P	B		S	B					B			B				
Martins et al. (2014) [85]	fMRI	Wisconsin Word Sorting Task	14	B	B														P		P				
Meinzer et al. (2012) [30]	fMRI	Category & letter fluency	14			S					P						P	P	P						
Rizio et al. (2017) [86]	fMRI	Naming with categorical & phonological distractors	20	S	S													S			S				
Rochon et al. (2010) [77]	fMRI	Semantic & rhyming judgment	10			P	P	P			P			P							B				
Rodriguez-Aranda et al. (2016) [61]	VBM & DTI	Category & letter fluency	42[a]	S	P		P		P	B				S			B		S				B	B	
Saykin et al. (1999) [78]	fMRI	Semantic & phonological decision	6	B	S						B						S								
Shafto et al. (2012) [79]	fMRI	Semantic & phonological lexical decision	16								S	S													
Sonty et al. (2003) [80]	fMRI	Synonym & homonym judgment	11		P		B	B			S	B													

van Hees et al. (2014) [87]	fMRI	SFA & PhCA treatment	14															S							
Vonk et al. (2019) [63]	Cortical thickness correlation	Category & letter fluency	505		B		S	B	B	B	B	S	S		P			B	S	S	B				
Wilson et al. (2009) [83]	fMRI	Reading irregular & pseudowords	9			P	P			P	B							B			B				
Zhang et al. (2013) [64]	VBM	Category & letter fluency	344			S		S		S		P					S								
Zhuang et al. (2016) [81]	fMRI	Semantic & rhyming judgment	20											S											
Semantic count (%):				2 (12%)	2 (12%)	2 (12%)	1 (6%)	1 (6%)	0 (0%)	1 (6%)	2 (12%)	3 (18%)	1 (6%)	3 (18%)	0 (0%)	0 (0%)	2 (12%)	2 (12%)	2 (12%)	1 (6%)	1 (6%)	0 (0%)	0 (0%)	0 (0%)	0 (0%)
Phonological count (%):				0 (0%)	2 (12%)	2 (12%)	3 (18%)	1 (6%)	2 (12%)	1 (6%)	2 (12%)	1 (6%)	0 (0%)	1 (6%)	1 (6%)	0 (0%)	1 (6%)	1 (6%)	3 (18%)	1 (6%)	1 (6%)	0 (0%)	0 (0%)	0 (0%)	0 (0%)
Both domains count (%):				3 (18%)	3 (18%)	1 (6%)	2 (12%)	3 (18%)	1 (6%)	3 (18%)	4 (24%)	2 (12%)	1 (6%)	0 (0%)	0 (0%)	0 (0%)	1 (6%)	4 (24%)	0 (0%)	0 (0%)	4 (24%)	0 (0%)	1 (6%)	2 (12%)	1 (6%)

**Note*. All regions are in the left hemisphere. [a]Sample size includes neurologically intact older adults and clinical participants because both groups were included in the analysis. [b]Sample size includes neurologically intact older adults and clinical participants because although some analyses were conducted within the separate groups, the only significant results relevant to the scoping review were in the combined sample. Across cells, S = found for semantic task only, P = found for phonological task only, B = found for both semantic and phonological tasks. Other abbreviations: AF = arcuate fasciculus, AG = angular gyrus, DSI = diffusion spectrum imaging, FC = fusiform cortex, fMRI = functional magnetic resonance imaging, HG = Heschl's gyrus, H/PG = hippocampus/parahippocampal gyrus, IFGop = inferior frontal gyrus-pars opercularis, IFGorb = inferior frontal gyrus-pars orbitalis, IFGtri = inferior frontal gyrus-pars triangularis, IFOF = inferior fronto-occipital fasciculus, Ins = insula, ILF = inferior longitudinal fasciculus, ITG = inferior temporal gyrus, LOC = lateral occipital cortex, MFG = middle frontal gyrus, MTG = middle temporal gyrus, PCA = principal components analysis, PhCA = phonological components analysis treatment, PG = precentral gyrus, PT = planum temporale, SFA = semantic feature analysis treatment, SFG = superior frontal gyrus, SMG = supramarginal gyrus, STG = superior temporal gyrus, TP = temporal pole, UF = uncinate fasciculus, VBCM = voxel-based correlational methodology, VBM = voxel-based morphometry, VLSM = voxel-based lesion symptom mapping.
