# Supplementary material for: Semantic and Phonological Brain Networks in Older Adults: A Systematic Scoping Review
Source: Brain Sci. 2026 Feb 25;16(3):252. doi: 10.3390/brainsci16030252 (PMC13024727; doi:10.3390/brainsci16030252)
Supplement: Supplementary file 1 [file brainsci-16-00252-s001.zip › brainsci-4138054-supplementary/TableS3_CLIN.pdf]

Supplementary Table S3. Region of interest results from studies involving clinical samples

Study	Method	Task	*N*	SFG	MFG	IFG orb	IFG tri	IFG op	PreG	Ins	STG	MTG	ITG	TP	HG	PT	H/PG	FC	SMG	AG	LOC	AF	ILF	IFOF	UF
Alyahya et al. (2018) [65]	VBCM	PCA of neuropsych battery	48								P	B	S	S	P	P		S	P	P	S	P	B		
Alyahya et al. (2020A) [67]	VBCM	PCA of neuropsych battery	46																P						
Alyahya et al. (2020B) [66]	VBCM	PCA of neuropsych battery	42								P	B	B		P	P		S		P	B	P	B	S	S
Baldo et al. (2006) [54]	VLSM	Category & letter fluency	48	P	P			P	P	B				S	S			S	B	B					
Biesbroek et al. (2021) [55]	VLSM	Category & letter fluency	1,231	B	B		S	B	B	B	B	B	B	B	B	B	B	S	B	B	B			B	
Boukrina et al. (2015) [75]	Lesion-deficit analysis	Picture & rhyme matching	11					P		P	P	P	P	P	P	P		P	P					P	P
Brambati et al. (2009) [82]	VBM	Reading irregular & pseudowords	66[a]								B	B		S				B	P	P					
Butler et al. (2014) [68]	VBCM	PCA of neuropsych battery	31							P	P	B			P	P						P	S	S	S
Chang et al. (2020) [56]	DSI	Category & phonemic fluency	86[a]																					B	B
Chouiter et al. (2016) [57]	VLSM	Category & letter fluency	191							B	B	B							B	B					
Ellfolk et al. (2014) [58]	VBM	Category & letter fluency	56[b]																						
Halai et al. (2017) [69]	VBCM	PCA of neuropsych battery	31							P	P	B	S		P	P		S	P			P	S	S	S
Halai et al. (2018) [70]	VBCM	PCA of neuropsych battery & naming errors	46							P	P	B	S	B	P		S	S	P						

Henry et al. (2012) [71]	VBM	PCA of language battery	15						P	P	P	S	S	S				S	P	S					
Pereira et al. (2009) [59]	VBM	Category & letter fluency	32									S	S	S			S								
Riello et al. (2022) [60]	volumetric analysis	Category & letter fluency	35										S												
Rodriguez-Aranda et al. (2016) [61]	VBM & DTI	Category & letter fluency	42 [a]	S	P		P		P	B				S			B		S				B	B	
Schmidt et al. (2019) [62]	VLSM	Category & letter fluency	85		P				P		S	S	S												
Schumacher et al. (2019) [72]	VBCM	PCA of neuropsych battery	38									P	B	S		P	S	B	P	P			B		
Stark et al. (2019) [84]	VLSM	Error types for picture naming & description	57								S	S	S	S				S		S	S		S	S	
Wilson et al. (2010) [88]	VBM	Error types for picture description	60																						
Woollams et al. (2018) [73]	VBCM	PCA of neuropsych battery	43		P	P	B	B	P	B	B	B	S	P	P	P	S	S	P			P	B		S
Zhao et al. (2018) [74]	VBCM	PCA of neuropsych battery	35									S							P	P		P			
Zhao et al. (2020) [89]	VBCM	PCA of neuropsych battery	70							P	P	B		S	P	P	S	S	P	P	S	P	S	S	S
Semantic count (%):				1 (4%)	0 (0%)	0 (0%)	1 (4%)	0 (0%)	0 (0%)	0 (0%)	2 (8%)	5 (21%)	9 (38%)	9 (38%)	1 (4%)	0 (0%)	5 (21%)	10 (42%)	1 (4%)	2 (8%)	3 (13%)	0 (0%)	4 (17%)	5 (21%)	5 (21%)
Phonological count (%):				1 (4%)	4 (17%)	1 (4%)	1 (4%)	2 (8%)	5 (21%)	6 (25%	8 (33%)	2 (8%)	1 (4%)	2 (8%)	8 (33%)	8 (33%)	0 (0%)	1 (4%)	11 (46%)	6 (25%)	0 (0%)	7 (29%)	0 (0%)	1 (4%)	1 (4%)
Both domains count (%):				1 (4%)	1 (4%)	0 (0%)	1 (4%)	2 (8%)	1 (4%)	5 (21%)	4 (17%)	10 (42%)	3 (13%)	2 (8%)	1 (4%)	1 (4%)	2 (8%)	2 (8%)	3 (13%)	3 (13%)	2 (8%)	0 (0%)	5 (21%)	3 (13%)	1 (4%)

Note. All regions are in the left hemisphere. [a]Sample size includes neurologically intact older adults and clinical participants because both groups were included in the analysis. [b]Sample size includes neurologically intact older adults and clinical participants because although some analyses were conducted within the separate groups, the only significant results relevant to the scoping review were in the combined sample. Across cells, S = found for semantic task only, P = found for phonological task only, B = found for both semantic and phonological tasks. Other abbreviations: AF = arcuate fasciculus, AG = angular gyrus, DSI = diffusion spectrum imaging, FC = fusiform cortex, fMRI = functional magnetic resonance imaging, HG = Heschl's gyrus, H/PG = hippocampus/parahippocampal gyrus, IFGop = inferior frontal gyrus-pars opercularis, IFGorb = inferior frontal gyrus-pars orbitalis, IFGtri = inferior frontal gyrus-pars triangularis, IFOF = inferior fronto-occipital fasciculus, Ins = insula, ILF = inferior longitudinal fasciculus, ITG = inferior temporal gyrus, LOC = lateral occipital cortex, MFG = middle frontal gyrus, MTG = middle temporal gyrus, PCA = principal components analysis/phonological components analysis treatment, PG = precentral gyrus, PT = planum temporale, SFA = semantic feature analysis treatment, SFG = superior frontal gyrus, SMG = supramarginal gyrus, STG = superior temporal gyrus, TP = temporal pole, UF = uncinate fasciculus, VBCM = voxel-based correlational methodology, VBM = voxel-based morphometry, VLSM = voxel-based lesion symptom mapping.
